# Conservation of cell-intrinsic immune responses in diverse nonhuman primate species

**DOI:** 10.26508/lsa.201900495

**Published:** 2019-10-24

**Authors:** Jenna M Gaska, Lance Parsons, Metodi Balev, Ann Cirincione, Wei Wang, Robert E Schwartz, Alexander Ploss

**Affiliations:** 1 Lewis Thomas Laboratory, Department of Molecular Biology, Princeton University, Princeton, NJ, USA; 2 Carl Icahn Laboratory, Lewis-Sigler Institute for Integrative Genomics, Princeton University, Princeton, NJ, USA; 3 Weill Cornell Medical College, Belfer Research Building, New York, NY, USA

## Abstract

Differences in immune responses across species can contribute to the varying permissivity of species to the same viral pathogen. Understanding how our closest evolutionary relatives, nonhuman primates (NHPs), confront pathogens and how these responses have evolved over time could shed light on host range barriers, especially for zoonotic infections. Here, we analyzed cell-intrinsic immunity of primary cells from the broadest panel of NHP species interrogated to date, including humans, great apes, and Old and New World monkeys. Our analysis of their transcriptomes after poly(I:C) transfection revealed conservation in the functional consequences of their response. In mapping reads to either the human or the species-specific genomes, we observed that with the current state of NHP annotations, the percent of reads assigned to a genetic feature was largely similar regardless of the method. Together, these data provide a baseline for the cell-intrinsic responses elicited by a potent immune stimulus across multiple NHP donors, including endangered species, and serve as a resource for refining and furthering the existing annotations of NHP genomes.

## Abstract (precis)

The transcriptomic response of diverse nonhuman primate (NHP) species to poly(I:C) is highly conserved, and this novel RNA sequencing dataset will help improve NHP genome annotations.

## Introduction

Many of the pathogens with a significant impact on human morbidity and mortality—such as HIV, hepatitis C virus, hepatitis B virus, and yellow fever virus—have a narrow host range limited to humans and select nonhuman primate (NHP) species (1). Broadly speaking, the host tropism of such viral pathogens can be determined by (i) the absence or incompatibility of (a) host factor(s) needed for part(s) of the viral life cycle; (ii) the presence of dominant restriction factors; and/or (iii) differences in immune responses. Understanding the molecular basis of host tropism can inform our understanding of intra- and interspecies transmission and aid in the generation of improved animal models and clinical therapies. Here, we analyzed how cell-intrinsic immune responses compare across humans and NHP species. As our closest evolutionary relatives, the tactics by which NHPs have evolved to confront pathogens and the corresponding ways those pathogens modulate host immune defenses—as highlighted by hotspots for positive selection in genes involved with immune/defense responses in humans and select NHPs ((2, 3, 4, 5), reviewed in reference 6)—could shed light on host range barriers pertinent to studying zoonotic infections and using NHPs as biomedical research models.

In the present study, we purposefully chose a cell type that could be easily collected from a wide array of multiple NHP species, was conducive to studies of cell-intrinsic immune responses and could be used for future generation of induced pluripotent stem cells to study host–pathogen interactions in desired cell lineages. The latter criterion would be especially advantageous for studying pathogens with both highly limited host and tissue tropism, such as hepatitis C virus, for which acquiring hepatocytes from NHPs can be extremely challenging for both ethical and financial reasons. Dermal fibroblasts (DFs) met all these requirements and could be obtained through biopsies or existing repositories. As an added advantage, we used only primary DFs which do not have the cell cycle dysregulation or disruption of immune signaling as observed for immortalized cells such as human hepatoma cell lines (7, 8).

**Figure 1.**
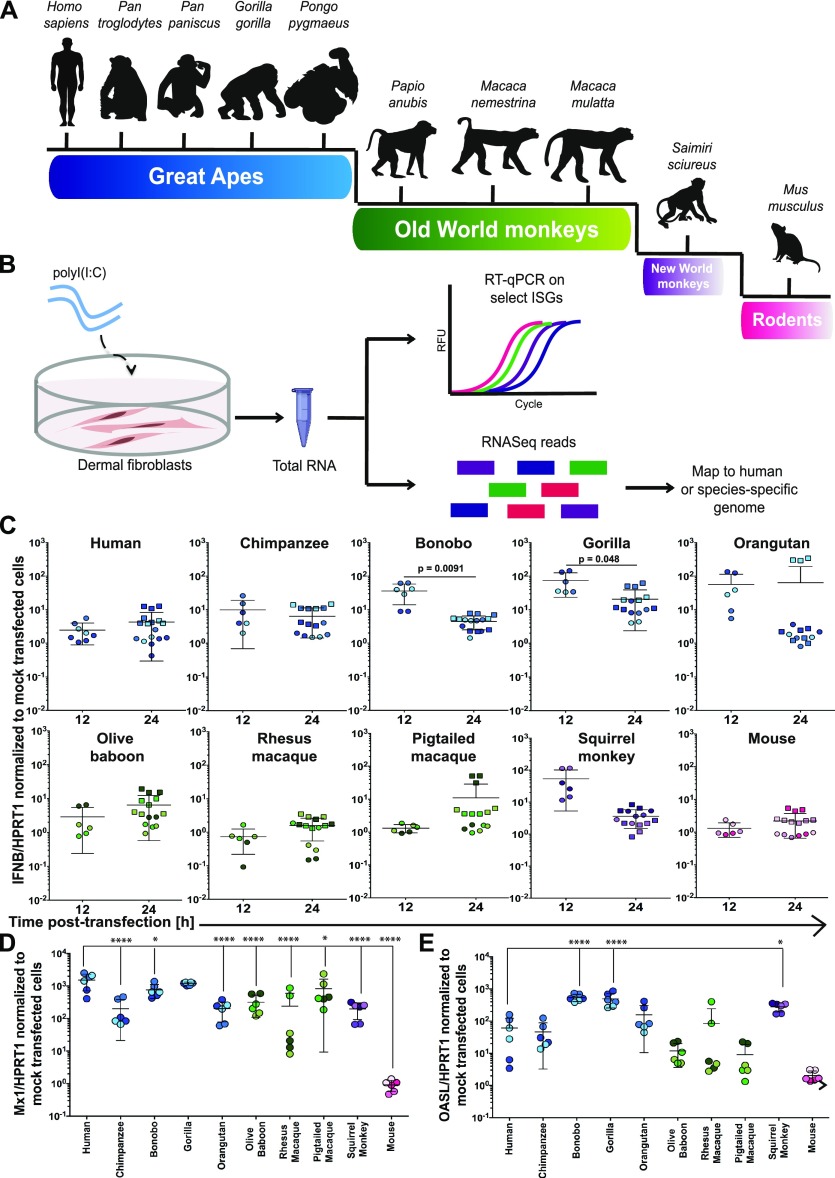
Comparing the cell-intrinsic immune response to poly(I:C) across multiple species. **(A)** Schematic representation of the phylogenetic relationship of species included in this study. **(B)** Overview of the experimental workflow. **(C, D, E)** Using RT-qPCR, *IFNβ* (C), *MX1* (D), and *OASL* (E) mRNA expression were assessed in primate and mouse primary DFs relative to the housekeeping gene *HPRT1* at 12 and/or 24 h after poly(I:C) (∼53 ng/cm^2^) or mock transfection. Values shown are the fold changes relative to mock-transfected cells. Each data point represents an individual well of cells and is colored by donor (see Fig S2 for the color code used). Circles represent data from 24-well experiments, whereas squares are from the transfections performed in six-well plates that underwent RNA-Seq. Data information: In (C, D, E), bars depict the mean with SD. Note that the lower error bar for the orangutan samples at both time points and the pig-tailed macaque at 24 h are missing because they approach a value of 0. Where *P* ≤ 0.05, the *P* value is indicated. In (C), an unpaired *t* test was used to compare timepoints, with Welch’s correction performed where the SD between time points differed by more than a factor of 2. In (D, E), an ordinary one-way ANOVA was performed followed by Dunnett’s multiple comparisons test with a single pooled variance and the human samples serving as the control against which all comparisons were made. *P ≤ 0.05; **P ≤ 0.01; ***P ≤ 0.001; ****P ≤ 0.0001. Source data are available for this figure.

We selected three donors from eight different NHP species covering ∼35 million years of evolution (Fig 1A and Table 1): great apes (*Pan troglodytes, Pan paniscus, Gorilla gorilla, *and* Pongo pygmaeus*), because of their close genetic relationship to humans but whose endangered status precludes their use in most scientific studies; three Old World monkey species (*Papio anubis, Macaca nemestrina, *and* Macaca mulatta*); and one New World species (*Saimiri sciureus*), which are all commonly used in biomedical research. As a more distant point in evolutionary time (ca. 65 million years since divergence), we also included mouse. For our initial studies comparing cell-intrinsic immunity in these DFs from diverse species, we aimed to provoke an immune response independent of a given species’ susceptibility and/or permissivity to a virus. Thus, we used polyinosinic:polycytidylic acid (poly(I:C)), a synthetic dsRNA analog that can be transfected into cells and potently stimulates an IFN-mediated response by resembling a pathogen-associated molecular pattern characteristic of RNA virus infections (Fig 1B) (9, 10, 11). This allowed us to compare the repertoire of cell-intrinsic responses available to DFs from each of these species unhindered by pathogen-mediated modulation that can occur as early as viral binding and entry.

**Table 1.** Known information for each DF donor.

	Common name	Species name	Classification	ID	Biopsy site	Sex	Age	Passage frozen
Donor 1	Pig-tailed macaque	*M. nemestrina*	Old World Monkey	AG07923	Skin, arm	Male	15 yr	2
Donor 2	Pig-tailed macaque	*M. nemestrina*	Old World Monkey	AG08490	Skin, arm	Male	22 yr	2
Donor 3	Pig-tailed macaque	*M. nemestrina*	Old World Monkey	PR00058	Skin, skin	Female	2 yr	2
Donor 1	Olive baboon	*P. anubis*	Old World Monkey	PR00033	Skin, skin	Male	3 yr	1
Donor 2	Olive baboon	*P. anubis*	Old World Monkey	PR00039	Skin, skin	Male	3 yr	2
Donor 3	Olive baboon	*P. anubis*	Old World Monkey	PR00036	Skin, skin	Male	3 YR	1
Donor 1	Squirrel monkey	*S. sciureus*	New World Monkey	AG05311A	Skin, skin	Female	UNKNOWN	14
Donor 2	Squirrel monkey	*S. sciureus*	New World Monkey	SQMA	Skin, stomach	Male	3 yr	4
Donor 3	Squirrel monkey	*S. sciureus*	New World Monkey	SQMB	Skin, stomach	Male	3 yr	4
Donor 1	Orangutan	*P. pygmaeus*	Great ape	AG06105	Skin, thoracolumbar junction	Female	26 yr	5
Donor 2	Orangutan	*P. pygmaeus*	Fibroblast	PR00054	Skin, skin	Male	4 yr	5
Donor 3	Orangutan	*P. pygmaeus*	Great ape	PR01109	Skin, leg	Female	40 yr	2
Donor 1	Gorilla	*G. gorilla*	Great ape	PR00230	Skin, skin	Female	37 yr	2
Donor 2	Gorilla	*G. gorilla*	Great ape	PR00573	Skin, inner thigh	Male	34 yr	2
Donor 3	Gorilla	*G. gorilla*	Great ape	PR00107	Skin, skin	Male	19 yr	3
Donor 1	Bonobo	*P. paniscus*	Great ape	PR00111	Skin, location unknown	Male	31 yr	4
Donor 2	Bonobo	*P. paniscus*	Great ape	PR00235	Skin, skin	Female	28 yr	5
Donor 3	Bonobo	*P. paniscus*	Great ape	PR00248	Skin, skin	Female	1 yr	2
Donor 1	Chimpanzee	*P. troglodytes*	Great ape	S004933	Skin, location unknown	Female	6 yr	2
Donor 2	Chimpanzee	*P. troglodytes*	Great ape	S003611	Skin, location unknown	Male	6 yr	1
Donor 3	Chimpanzee	*P. troglodytes*	Great ape	S003649	Skin, location unknown	Male	9 yr	1
Donor 1	Rhesus macaque	*M. mulatta*	Old World Monkey	AG08308	Skin, arm	Male	1 yr	1
Donor 2	Rhesus macaque	*M. mulatta*	Old World Monkey	AG08312	Skin, arm	Female	1 yr	1
Donor 3	Rhesus macaque	*M. mulatta*	Old World Monkey	AG08305	Skin, arm	Male	1 yr	1
Donor 1	Human	*Homo sapiens*	Great ape	NHDF	Skin, location unknown	Female	1 yr	7
Donor 2	Human	*H. sapiens*	Great ape	NHDF AF	Skin, location unknown	Male	1 yr	6
Donor 3	Human	*H. sapiens*	Great ape	NHDF R06	Skin, location unknown	Male	1 yr	5
Donor 1	Mouse	*Mus musculus* (C57/BL6)	Rodentia	C57A	Skin, abdomen	Male	8–10 wk	1
Donor 2	Mouse	*M. musculus* (C57/BL6)	Rodentia	C57B	Skin, abdomen	Male	8–10 wk	1
Donor 3	Mouse	*M. musculus* (C57/BL6)	Rodentia	C57C	Skin, abdomen	Female	8–10 wk	1

We subsequently analyzed the transcriptomic profile of these cells, generating data in response to a mimic of viral infection in the broadest panel of NHP species compiled to date. Although the evolution of select factors in response to host–pathogen conflict has been explored (12, 13), there has not been a comprehensive analysis of cell-intrinsic responses in nonimmune cells across a diverse range of NHPs. Much of comparative primate immunology focuses on the major cellular constituents of innate and adaptive immunity (14, 15, 16, 17, 18, 19) and highly variable genetic loci such as those encoding MHC class I molecules (20, 21, 22), immunoglobulins (23), and MHC class I–related (MR1) molecules (24). Furthermore, such studies often use a limited number of NHP species and focus on differences at the genetic level versus expression. To our knowledge, the closest study to ours examining the functional consequences of differences and similarities in innate immunity in NHPs stimulated primary monocytes from chimpanzee, rhesus, and human with LPS (25). Two more recent studies also provided insights into cell-intrinsic immunity but on a much larger evolutionary scale, including human or human and rhesus macaque as the only primates (26, 27).

The immense transcriptomic dataset we have acquired and analyzed in the course of this work fulfills a need for not only better understanding cell-intrinsic immunity in our closest evolutionary relatives but also providing additional sequencing information from NHP species, including those that are endangered. In this study, we mapped the data from each species both to the human genome and to the species-specific genomes, creating the added opportunity of comparing these two approaches in parallel. Overall, we observed a high level of conservation in the functional responses of the NHP DFs to poly(I:C) and found that with the current state of genome annotations, the percent of reads assigned to a genetic feature were largely similar between the two mapping methods. We anticipate that making these data available will enhance the continued efforts to more fully annotate NHP genomes and to aid in the identification of novel transcripts unobserved in humans. In addition, we believe these unique data will greatly facilitate evolutionary analyses, especially in the area of comparative immunology.

## Results and Discussion

### Expression of select interferon-stimulated genes (ISGs) in response to poly(I:C) varies across diverse species

**Figure S1.**
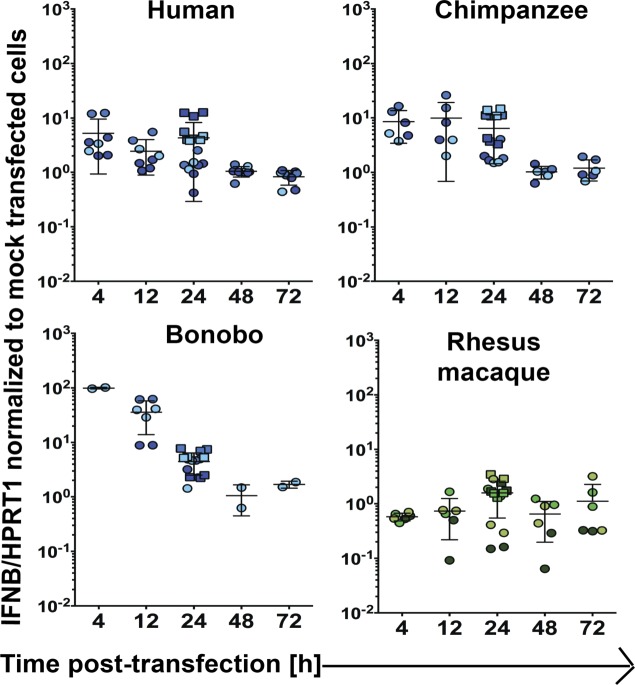
Extended kinetics of IFNβ expression in select donors. For select donors, time points were collected in addition to those at 12 and 24 h post-transfection with poly(I:C) (∼53 ng/cm^2^) or mock transfection as shown in Fig 1. Each data point represents an individual well of cells and is colored by donor (Fig S2). Circles represent data from 24-well experiments whereas squares are from the transfections performed in six-well plates that underwent RNA-Seq. Bars depict the mean with SD.

**Figure S2.**
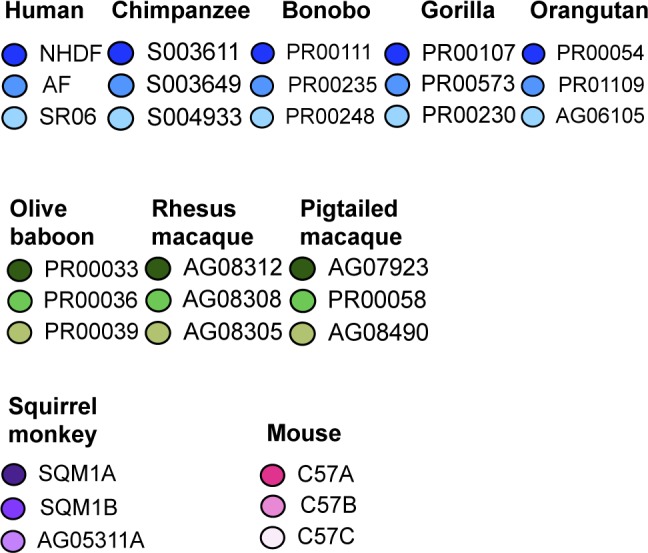
Color coding system used throughout the article for the individual donors.

Triggering innate immune sensing pathways characteristically provokes the induction of hundreds of ISGs, which collectively create an antiviral state. However, it remains unclear how similar such responses are across NHP species. To initially test whether poly(I:C) would elicit a cell-intrinsic response in the DFs we had from 10 different species (each represented by three donors), we first assessed transcription via RT-qPCR of *IFNβ* at 12 and 24 h post-transfection and two well-characterized ISGs, myxoma resistance protein 1 (*MX1*) and 2′-5′-oligoadenylate synthetase-like (*OASL*, *Oasl1* in mice), 24 h post-transfection using species-specific primers (Figs 1C–E, S1, and S2). *IFNβ* mRNA had on average less than a 10-fold change in expression relative to mock-transfected cells for most species (Fig 1C). However, bonobo, gorilla, orangutan, and squirrel monkey did exceed this for at least one of the time points tested. Bonobo and gorilla had significantly higher *IFNβ* expression at 12 h compared with 24 h, whereas the opposite trend was observed for rhesus macaque. The two ISGs examined demonstrated far greater fold changes relative to mock-transfected cells, underscoring the prominence of the downstream response to IFN at 24 h post-transfection. All species, with the exception of mouse, had at least an average 100-fold increase relative to mock in *MX1* mRNA, although there was marked variation amongst rhesus donors. Average levels of *OASL* mRNA were also elevated across the primate donors, albeit with greater donor variation, but all three mouse donors still displayed minimal change. The donor–donor variation we observed was not surprising as the NHP fibroblasts were acquired from outbred donors, with at least one male and one female represented for each species. As these data represent only a small sample of the hundreds of ISGs (28, 29, 30) whose expression could be changing after poly(I:C) transfection, we performed RNA-Seq on total mRNA isolated from these DFs 24 h post-transfection for a more comprehensive view. Unlike recent studies that looked earlier at 4 h post-immune stimulation (26, 27), we wanted to analyze effects further downstream as the cell-intrinsic response was amplified over time.

### Using species-specific genomes increases read alignment but to regions with no assigned features

**Figure S3.**
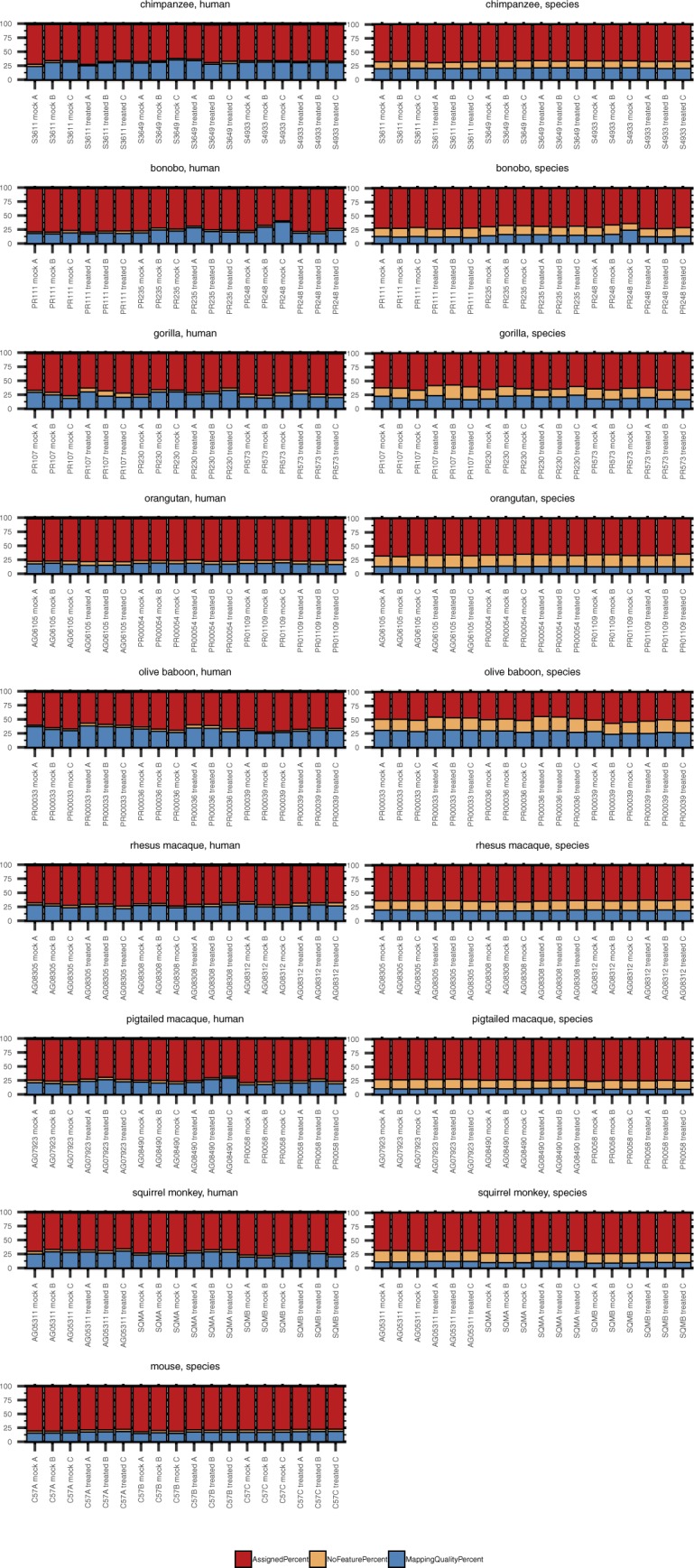
The percentage of RNA-Seq reads aligned and assigned for each species after mapping to either the human genome or the species-specific genome. Total RNA was extracted from DFs 24 h after poly(I:C) or mock transfection followed by cDNA library preparation and sequencing. Samples originated from nine species, as shown grouped here, with three donors per species and each donor transfected in triplicate with either poly(I:C) or mock transfected. The donor ID and transfection condition (“mock” or “treated” with poly(I:C)) are indicated on the X axis of each grouped species plot. The NHP species reads were mapped to the human genome (“human method”) or their species-specific genome (“species method”) (see Table 2) and then the percent alignment and assignment to each genome determined by RNA STAR and featureCounts, respectively. As indicated in the figure legend, bars in shades of red indicate percent alignment and blue percent assignment. The darker shade of each is for the mapping performed with the human genome and the lighter shade the mapping performed with the species-specific genome for each NHP species. Source data are available for this figure.

In working with such a diverse array of species, one challenge was choosing the genomes upon which to map the NHP-derived RNA-Seq reads. Since the genomes for human and mouse are well-annotated and species-specific resources available for downstream analyses, we felt confident in aligning reads from these species to their respective genomes. However, for the NHP species, to make our analysis more thorough, we used two approaches in parallel: mapping all primate-derived reads to the human genome as previously performed (31, 32, 33, 34) (hereby referred to as “human method”) or to their respective genomes as they currently exist on Ensembl (Table 2) (“species method”). Human and mouse reads averaged above 90% alignment and ∼80% assignment. For the great ape species examined, the percent alignment was generally the same regardless of the genome used, exceeding 85% (Fig S3). For the Old World monkeys, there was a small but noticeable decline when mapping to the human genome, with percent alignment close to 85% for the macaques and dipping under 80% for samples from olive baboon. Aligning reads from each of these species to their respective genomes increased these values, especially for rhesus and pig-tailed macaque, which approached 90% and 95%, respectively. Moving further out evolutionarily, squirrel monkey had the lowest percent alignment to the human genome, but still most reads did map, centering around 70%. Not surprisingly, more than 90% of reads were aligned when the squirrel monkey genome was used.

**Table 2.** Reference genomes used.

Species	Reference genome	Last genebuild update
Orangutan	ppyg2 (Ensembl 93)	August 2012
Gorilla	gorGor4 (Ensembl 95)	January 2018
Pig-tailed macaque	Mnem_1.0 (Ensembl 95)	January 2018
Rhesus macaque	Mmul_8_0_1 (Ensembl 95)	October 2016
Bonobo	panpan1.1 (Ensembl 95)	January 2018
Chimpanzee	Pan_tro_3.0 (Ensembl 95)	January 2018
Olive baboon	papAnu2 (Ensembl 95)	January 2018
Squirrel monkey	SaiBol1.0 (Ensembl 95)	January 2018
Human	GRCh38 (Ensembl 96)	November 2018
Mouse	GRCm38 (Ensembl 85)	May 2016

Despite these variations in alignment depending on the reference genome used, the percent of reads assigned to a genome feature by the “human method” tended to be higher (up by ∼5–6%) or comparable with that of the “species method.” Only for some chimpanzee, pig-tailed macaque and squirrel monkey samples did the species mapping improve the percent assignment (once more up by ∼5–6%). Orangutan and olive baboon demonstrated the greatest differences, with the “species method” as much as ∼10 or 15 percentage points lower, respectively, than the “human method.”

**Figure S4.**
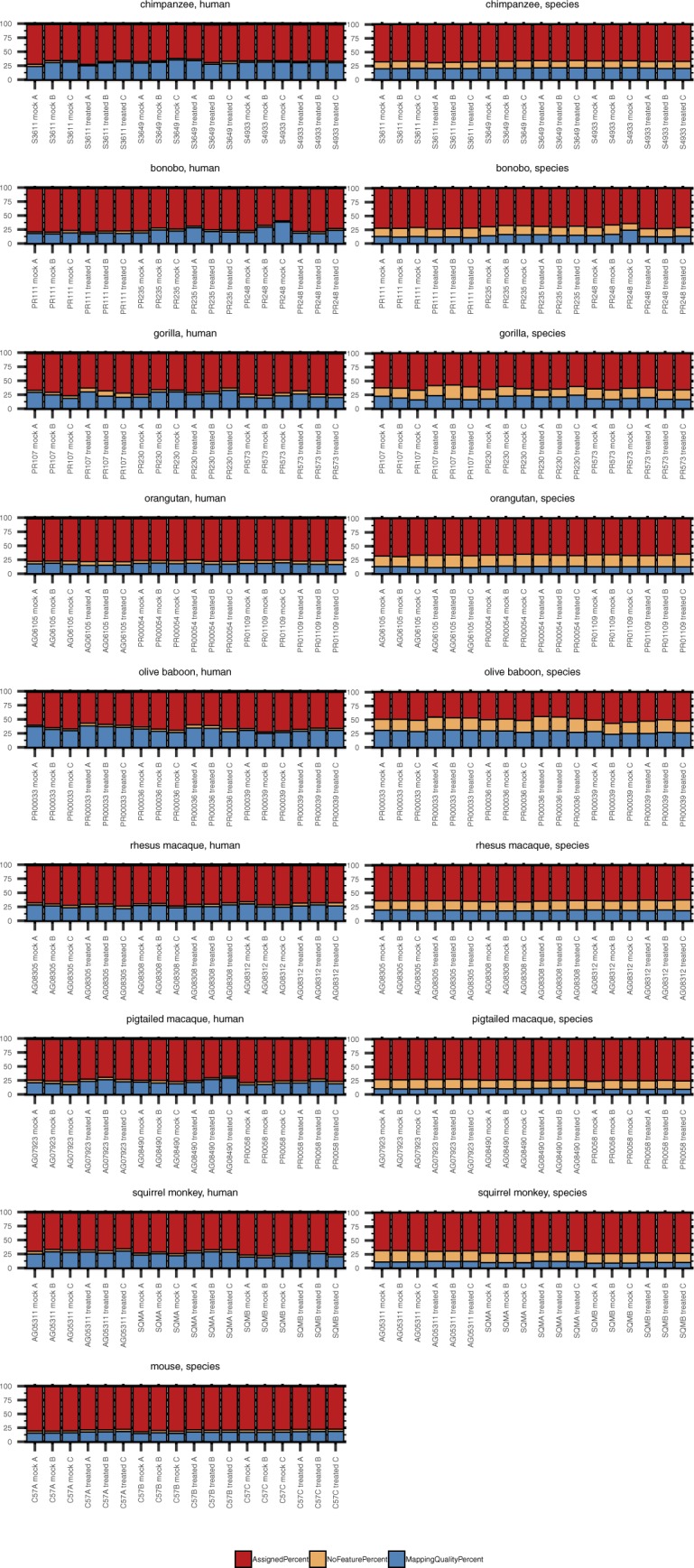
Comparison of feature assignment between the human and species mapping methods. Total RNA was extracted from DFs 24 h after poly(I:C) or mock transfection followed by cDNA library preparation and sequencing. Each grouped bar graph represents an NHP species, with results from reads aligned to the human genome shown in the left column and those from reads aligned to the species-specific genome (see Table 2) in the right column. The donor ID and transfection condition (“mock” or “treated” with poly(I:C)) are indicated on the X axis of each plot. featureCounts was used to determine the percent of reads that were assigned to an existing genome feature (red), the percent that mapped but with no known feature to assign the reads to (orange), and the percent that were unassigned because of low mapping quality (blue). Source data are available for this figure.

Taking a closer look at the relationship between the percent of reads assigned to a genome feature and the mapping method, we observed that the benefit of the “species method” over the “human method,” whereby the percentage of reads failing to be assigned a feature because of poor mapping quality declined, was less advantageous than expected as an increased percentage of the reads that mapped were to regions with no assigned feature (Fig S4). Notably, this was consistent across NHP species, irrespective of evolutionary relationship to humans. Thus, the percentage of reads assigned a feature was generally comparable between the two mapping methods or better by the “human” method. The NHP genome annotations as they currently exist resulted in on average anywhere from ∼12–22% of reads mapping to sequence with no assigned feature (for comparison, <5% of human and mouse reads fell in this category after alignment to their respective genomes). In contrast, less than 5.5% of reads fell in this category when using the “human method.” Identifying what these unassigned features might be beyond the extensive homologous sequence searching across existing genomes already performed by Ensembl is outside the scope of this article. These data highlight the weaknesses of the current NHP genome annotations and the core difficulty in performing comparative analyses, as the more divergent, species-specific sequences will still be missed regardless of the genome used. We hope that sequencing datasets like ours from multiple donors of multiple NHP species will aid in improving such annotations.

### Species cluster by evolutionary relationship when looking at overall transcriptomic response

**Figure S5.**
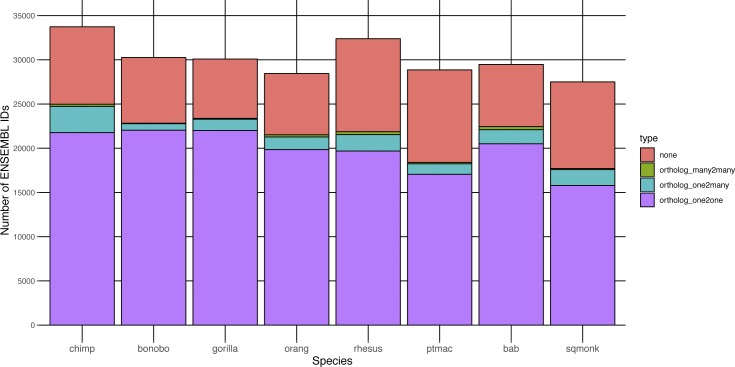
Types of human orthologs found across NHP species. The ENSEMBL gene IDs for each NHP species under examination were gathered and the human ortholog, if any, retrieved from bioMart along with the ortholog “type” (none, one-to-one, one-to-many, or many-to-many). The number of NHP ENSEMBL gene IDs falling into each of these ortholog “type” categories are shown here in a stacked bar graph, with each column representing a different species and the colors within each column representing a different ortholog type. chimp, chimpanzee; orang, orangutan; rhesus, rhesus macaque; ptmac, pig-tailed macaque; bab, olive baboon; and sqmonk, squirrel monkey.

**Figure 2.**
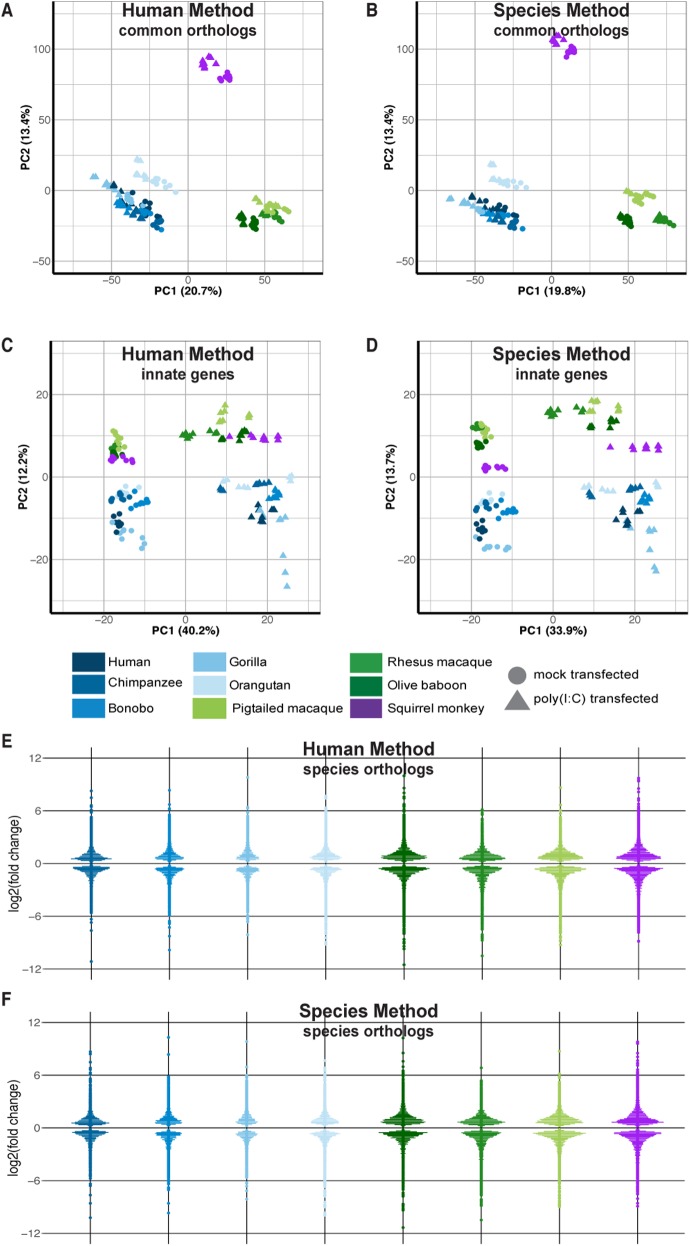
Overall transcriptomic responses to poly(I:C) cluster by species’ phylogenetic relationships and innate immune genes by treatment condition. **(A, B, C, D)** RNA sequencing reads were aligned to the human genome (A, C) or the respective NHP species from which the samples were derived (B, D). The resultant counts from either mapping method were then limited to genes with a one-to-one human ortholog (based off Ensembl ID) across all species, resulting in ∼11,000 genes (referenced as “common orthologs” in the main text). For counts mapped to the NHP genomes, the human ortholog Ensembl ID was used for all subsequent analysis for ease of comparison. These counts were normalized using DESeq2 default options and finally transformed using the regularized log_2_ function in DESeq2. **(A, B, C, D)** The first two components of the PCA are depicted here for either all ∼11,000 genes (“common orthologs”) (A, B) or for a subset of these genes which overlapped with those found in the database InnateDB (“innate genes”) (C, D). Each point represents an individual donor and is colored by species as shown in the figure legend, with shades of blue indicating great ape species, shades of green Old World Monkeys, and purple the single New World Monkey used in this study. Circles indicate mock-transfected samples and triangles poly(I:C)-transfected samples. **(E, F)** The counts from mapping to either the human or the species-specific genome were limited on a species-by-species basis to the Ensembl IDs that had a one-to-one human ortholog (referenced as “species orthologs” in the main text). The counts of these genes were then processed in DESeq2 to determine DGE, specifically comparing the DGE profile of each NHP species (poly(I:C)-transfected versus mock-transfected) to that of human. Thus, genes from NHP species that significantly differed from human in their response to poly(I:C) could be determined and are shown here as pseudo-violin plots from either the human genome (E) or the species-specific genome (F) mapping method. Each point represents an individual gene and is colored by species as shown in the figure legend. Data information: in (E, F), the differentially expressed genes shown have *P*_adj_ ≤ 0.05.

Across all NHP species examined, most genes had a one-to-one human ortholog, with the second largest category of genes those without a known ortholog (Fig S5). For both the “human” and the “species” methods, we first limited the resultant mapped read counts to only those genes that had one-to-one orthologs across all species (“common orthologs”), resulting in a common denominator of 11,677 genes (Supplemental Data 1). Using such an approach, we were able to directly compare all eight NHP species with one another, finding that by principal component analysis (PCA), the samples clustered according to the evolutionary relationship of the species, with great apes, Old World monkeys, and the single New World monkey species forming their own distinct groupings regardless of whether they were mapped by the “human” or the “species” method (Fig 2A and B). For both methods, the orangutan samples formed a group slightly removed from the other great ape species. However, only by the “species” method did we observe resolution of the Old World monkey species into three distinct groups unlike by the “human” method. This could be, at least in part, attributed to the larger differences in percent assignment of reads, especially between olive baboon and the two macaques, when using the “species” method (Fig S3).

Supplemental Data 1. List of human Ensembl IDs for genes which have a one-to-one ortholog across all NHP species (“common orthologs”).

### Innate immune genes strongly up-regulated by poly(I:C) transfection across species

**Figure S6.**
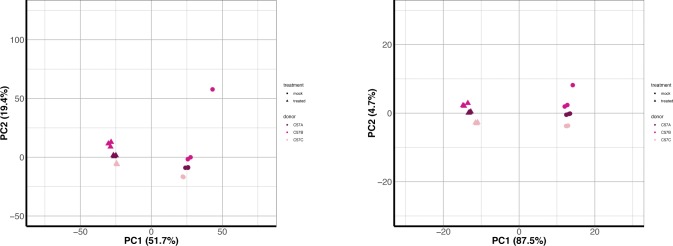
PCA plots of mouse samples. Reads from the murine samples were aligned to the mouse genome and the species annotations used for all analysis. These counts were normalized using DESeq2 default options and finally transformed using the regularized log_2_ function in DESeq2. The first two components of the PCA are depicted here for either all genes (left) (not limited by any type of orthologs with human) or for a subset of innate immune response genes from the database InnateDB (“innate genes”) (right). InnateDB has both human and murine genes listed, so whereas the primate samples used the human genes, here we were able to use the murine genes. Each point represents an individual donor, colored as shown in the legend. Circles indicate mock-transfected samples and triangles poly(I:C)-transfected samples.

As we had transfected the cells with poly(I:C), a synthetic dsRNA mimic known to induce cell-intrinsic immune responses, we wanted to focus further on known innate immunity genes and how their expression compared across these primate species. We limited our “common orthologs” output to a manually curated set of genes involved with innate immunity, InnateDB (35). This database includes 988 distinct gene symbols for human, 656 of which fell into our “common orthologs” set as one-to-one orthologs across all species (Supplemental Data 2). Examining these genes across our samples by PCA, we now observed clear separation of samples based on whether they had been transfected with poly(I:C) (Fig 2C and D, mouse shown in Fig S6), indicating the strong contribution of treatment in differentiating samples based off these genes. Within the treated and mock samples, the great ape samples did cluster separate from the monkey species, although mapping by the “species method” did result in greater separation between Old and New World monkey species. The squirrel monkey samples, our only New World monkey species under consideration, were closer than the Old World monkeys to the great ape cluster, especially after the “species method,” suggesting higher similarity of these genes’ expression with the great apes even though they are more distantly related.

Supplemental Data 2. The 656 genes from the InnateDB database that were among the “common orthologs” listed in Supplemental Data 1.

**Figure S7.**
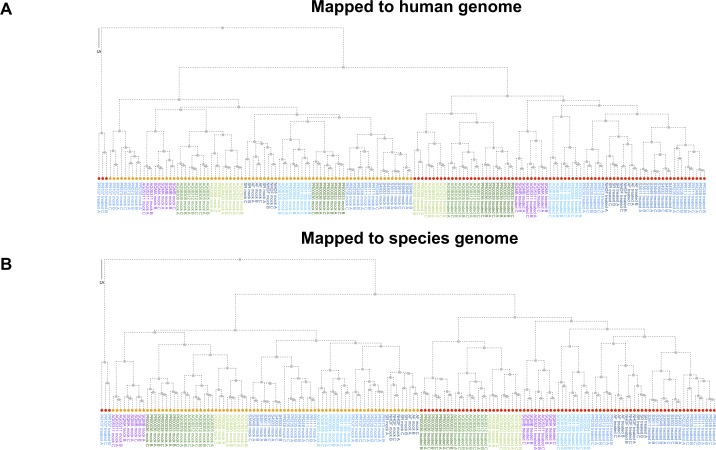
Cluster dendrogram of samples based off read counts for InnateDB genes with a one-to-one human ortholog across all NHP species examined. Hierarchical clustering was determined for the sample–sample distances for the normalized, regularized log_2_-transformed counts that were used to generate Fig 2 PCA plots of InnateDB genes with a one-to-one human ortholog across all NHP species. **(A, B)** The human method results are shown in (A) and the species method results in (B). Each tip label indicates the donor ID, treatment condition and replicate number. The tip labels are colored according to the larger classifications of the species (i.e., great ape or Old World Monkey) as used throughout the article. The tips themselves are colored based on treatment condition (orange for mock-transfection and red for poly(I:C)-transfection).

We examined the sample-to-sample distances of these same data to see if we still observed a closer relationship between the great apes and squirrel monkey when not limited to just the first two principal components. Hierarchical clustering of the sample-to-sample distances was highly similar regardless of mapping method, clearly distinguishing between mock and poly(I:C)-transfected samples as well as the distinct nature of poly(I:C)-transfected samples from gorilla donor PR00107 (Fig S7). In addition, despite the PCA plot for the “human method” showing less separation between the New and Old World monkeys, we were able to confirm the greater similarity of the squirrel monkey samples to the great apes in regards to this gene set (Fig S7). Although squirrel monkey was the most distant of our NHP species from humans, the clustering of this species with the great apes, instead of out beyond the Old World monkeys, suggests strong conservation of the response in these innate immune genes after poly(I:C) transfection. However, the exact basis for these observations cannot be determined, especially without examining more New World monkey species to see if this is broadly applicable.

### Determining the NHP response to poly(I:C) distinctive from that of human

**Figure S8.**
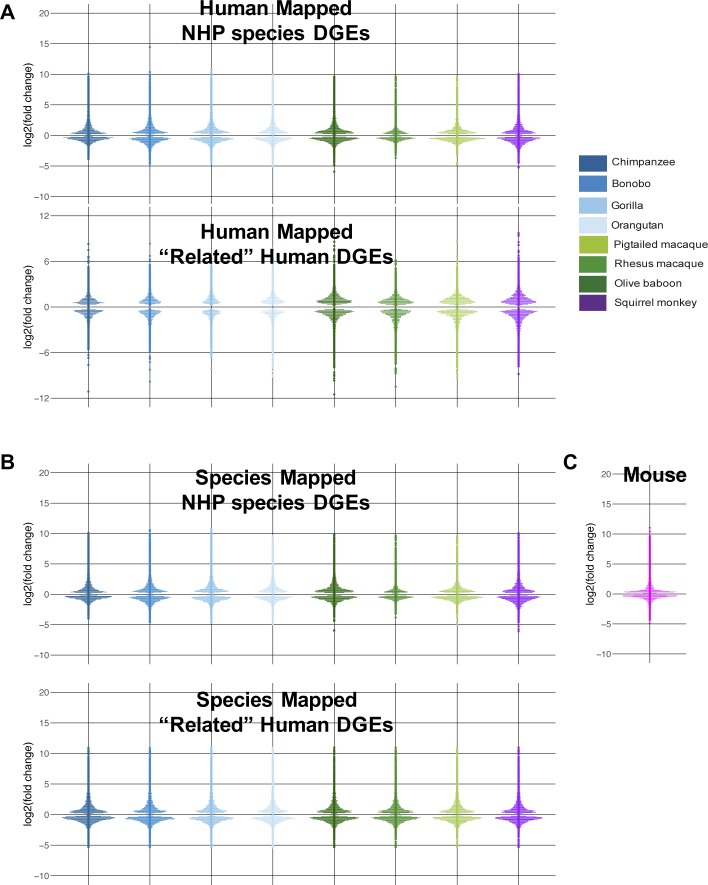
Summary of DGE in diverse species after poly(I:C) transfection. poly-A–containing RNA transcripts isolated from DFs mock-transfected or transfected 24 h with poly(I:C) were analyzed by RNA-Seq. **(A, B)** Shown are pseudo-violin plots of the distribution of differentially expressed genes (depicted as the log_2_(foldchange) in expression between poly(I:C)-transfected samples and mock samples) in human and NHP samples after mapping RNA-Seq reads to either the human (A) or species-specific (B) genomes. In both cases, genes were limited on a species-specific basis to those that have a one-to-one human ortholog. Hence, the top panel of (A) and (B) show the expression of genes from the NHP species and the lower panel that of the “related” human DGEs, which were limited to the same genes as those for the corresponding NHP species. Each pseudo-violin plot is colored according to species as shown in the figure legend. **(C)** Pseudo-violin plot of the DGE for the RNA-Seq reads from mice, which were all aligned to the mouse genome. Data information: in (A, B, C), the differentially expressed genes shown have *P*_adj_ ≤ 0.05.

Although informative for broader analyses, using the genes with a one-to-one human ortholog across all eight species was far more limiting than if we did so on a species-by-species basis (referred to as “species orthologs”). Such an approach substantially increased the possible number of Ensembl IDs from 11,766 to 15,793-22,040 (Table 3; note that after determining the one-to-one human ortholog for a given NHP gene, the human Ensembl ID was used). In looking at the differential gene expression (DGE) profiles (poly(I:C)-transfected versus mock-transfected) for each species using “species orthologs,” we observed similar distributions, with more genes increasing versus decreasing in expression after poly(I:C) transfection compared with mock-transfected cells (Fig S8, and Supplemental Data 3 and 4). Although the range of expression for up-regulated genes was larger compared with down-regulated genes, most genes had a |log_2_(fold change)| <3 relative to mock-transfected cells. Because we were most interested in how the NHP species compared with human, we used the latter as our baseline to assess DGE for each species (*P*_adj_ ≤ 0.05), finding on average ∼2,800 genes differentially expressed compared with human among the great ape species and ∼4,800 among the monkeys (Fig 2E and F, and Supplemental Data 5).

**Table 3.** Number of Ensembl ID annotations for each NHP species that is listed as having a one-to-one human ortholog.

Species	Number of one-to-one orthologs with human
Chimpanzee	21,766
Bonobo	22,040
Gorilla	21,998
Orangutan	19,843
Olive baboon	20,506
Rhesus macaque	19,680
Pig-tailed macaque	17,058
Squirrel monkey	15,793

Supplemental Data 3. These files are the DGE (poly(I:C)–transfected versus mock-transfected) outputs for each of the species after mapping to the human genome and limiting the genes for each NHP species to those that have a one-to-one human ortholog. The Ensembl IDs and gene information used are that of human. For each NHP species, there is an accompanying human DGE profile corresponding to the same limited list of one-to-one orthologs. Please see the README file for this dataset for further details.

Supplemental Data 4. These files are the DGE (poly(I:C)–transfected versus mock-transfected) outputs for each of the species after mapping to the species-specific genome and limiting the genes for each NHP species to those that have a one-to-one human ortholog. The Ensembl IDs and gene information used are that of human. For each NHP species, there is an accompanying human DGE profile corresponding to the same limited list of one-to-one orthologs. Please see the README file for this dataset for further details.

Supplemental Data 5. These files represent the DGE of each NHP species relative to the human DGE profile in response to poly(I:C) treatment. As mentioned in the main text, the genes for each NHP species were limited to those that have a one-to-one human ortholog. The Ensembl IDs and gene information used are that of human. Please see the README file for this dataset for further details.

**Figure 3.**
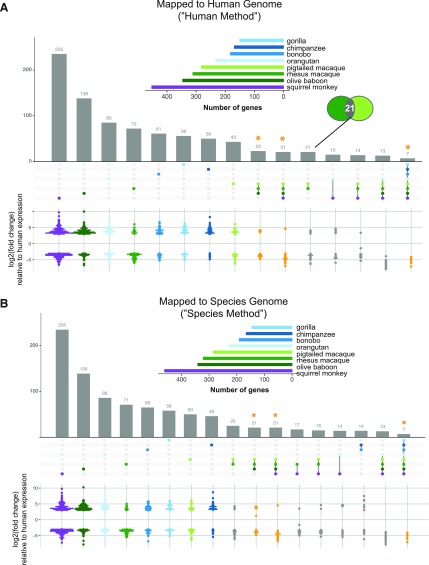
Overview of genes in NHP species and subgroups that differ markedly in their response to poly(I:C) compared with that of human. **(A, B)** For the genes shown in Fig 2E and F, the RNA-Seq reads were mapped to either the human genome (A) or the species-specific genome (B). The resultant gene counts from each mapping method were then limited on a species-by-species basis to those for which there is a one-to-one human ortholog. DGE upon poly(I:C) transfection for each of the NHP species was then assessed relative to that of human. The vertical bar graph in the upper panel displays the number of genes which were differentially expressed (*P*_adj_ ≤ 0.05, |log_2_(fold change)| ≥ 3) by a given species or group of species, as indicated by the matrix below the graph, compared with human. Each bar, thus, represents a unique group of genes with the matrix beneath indicating in which species these genes are expressed, similar to what is represented by the “intersection” of circles in a Venn diagram (as indicated by the inset Venn diagram in (A)). For ease of presentation, only the intersections with 14 or more genes are shown, except for the intersection of seven genes that differ from human in their expression upon treatment in all the species. The points in the matrix correspond to the different species (color-coded) from which DFs were sourced, with shades of blue indicating great ape species, shades of green Old World Monkeys and purple the single New World Monkey used in this study. The inset horizontal bar graph shows the total number of differentially expressed genes for each species based on the cutoff used (*P*_adj_ ≤ 0.05, |log_2_(fold change)| ≥ 3). The pseudo-violin plot below the matrix summarizes the differential log_2_(fold change) expression relative to human for the genes included in the corresponding vertical bar. For genes expressed by multiple species (i.e., all except the first eight columns), the average log_2_(fold change) across species for each gene was used. For the first eight columns of genes unique to each species, the dots are colored in accordance with the horizontal bar graph. An orange asterisk with corresponding orange points in the pseudo-violin plot highlights the gene groups of particular interest that are in common across larger NHP groupings: all Old World monkeys, all monkeys, or all species.

**Figure S9.**
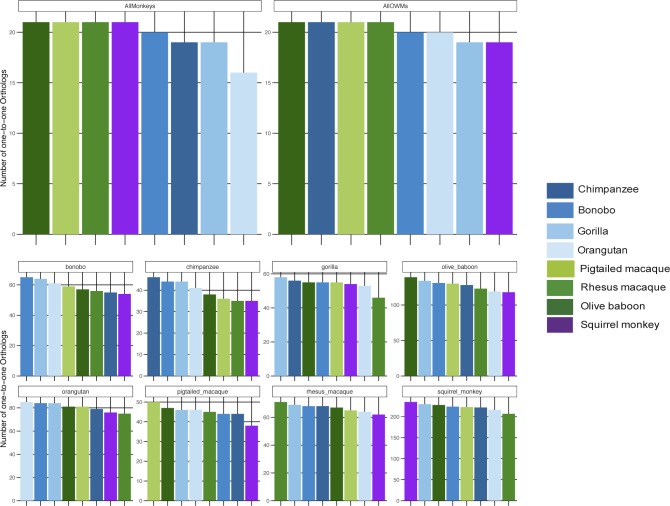
Most genes that are “uniquely” (i.e., only observed in one species or in a certain subset of species) significantly differentially expressed (*P*_adj_ ≥ 0.05) compared with human in response to poly(I:C) have a one-to-one human ortholog in the other NHP species. These data correspond with that shown in Fig 3 and delineates whether the genes that were found to be “unique” in their differential expression after poly(I:C) treatment compared with human was due to those genes having a one-to-one human ortholog only in that particular species or species group. Because the determination of one-to-one orthologs was done on a species-by-species basis before the analysis summarized in Fig 3, some genes could be found as “unique” to a species just because there was no one-to-one human ortholog in any of the other species. The upper left panel shows the “unique” genes amongst all the monkey species, the upper right panel that for the Old World monkeys and the bottom two rows those that were “unique” to individual species. The columns are colored based on the species as done throughout the rest of the article and as shown in the figure legend. The first column for each grouped plot (or columns in the case of the upper two panels) is that of the species given in the plot title. The y axis shows the number of genes that have a one-to-one human ortholog for each species. The maximum value is that of the first column (or group of columns for the upper two panels) and corresponds to the number of genes contained within that intersection as shown in Fig 3.

To find genes in each species with markedly different expression in response to poly(I:C) from that of human, we limited these DGEs to those with a |log2(fold change)| ≥ 3 and then compared their occurrence across the species. By either mapping method, more genes overall were differentially expressed compared with human in accordance with the evolutionary distance of the species from human (Fig 3, horizontal bar graphs). Furthermore, ∼80–89% of these genes overlapped between the two mapping methods (Table 4). A fraction of these genes were unique to each NHP species (Fig 3, first eight columns), with proportionally the greatest in two of the species more distantly related to human: squirrel monkey and olive baboon. By either mapping method, most genes unique to a given species had higher expression relative to human except for rhesus macaque, where the opposite trend was observed, and squirrel monkey and pig-tailed macaque, which each had an almost equal number of genes with higher and lower expression (Fig 3, pseudo-violin plots). In all cases, most genes found as uniquely different from human in a given species or species grouping were one-to-one orthologs across all species (Fig S9). Thus, these genes’ status as “unique” was not simply a result of their exclusion by our “species orthologs.”

**Table 4.** Overlap of genes between mapping methods for each NHP species significantly different in their transcriptomic response to poly(I:C) compared with that of human.

NHP species being compared with humans	No. of genes from mapping to species-specific genome^a^	No. of genes from mapping to human genome^a^	No. of genes in common between two mapping methods	Percentage of species genome–mapped genes in common between methods	Percentage of human genome–mapped genes in common between methods
Bonobo	191	182	153	80.10	84.07
Chimpanzee	166	169	143	86.14	84.62
Gorilla	146	150	127	86.99	84.67
Olive baboon	341	346	276	80.94	79.77
Orangutan	227	233	194	85.46	83.26
Pig-tailed macaque	285	282	252	88.42	89.36
Rhesus macaque	321	311	276	85.98	88.75
Squirrel monkey	461	452	396	85.90	87.61

a Genes in treated-versus-mock NHP samples that differed from treated-versus-mock human samples by abs[log_2_(foldchange)] ≥ 3, *P*_adj_ ≥ 0.05

In addition, of the possible combinations of species with common genes differentially expressed compared with human, we focused on NHP groupings of evolutionary significance (Fig 3, columns with orange asterisks): Old World monkeys, all monkey species, and all species (there were no genes common to all the great apes). For these genes, the average differential expression relative to human was determined for the species in each grouping and tended by either mapping method to be lower than that of human (Fig 3, pseudo-violin plots). Our finding that of the 3,000–6,000+ genes differentially expressed (*P*_adj_ ≤ 0.05) upon poly(I:C) treatment in the NHPs <500 were differentially expressed compared with human at our cutoff highlights the similarity of these species with human.

**Figure S10.**
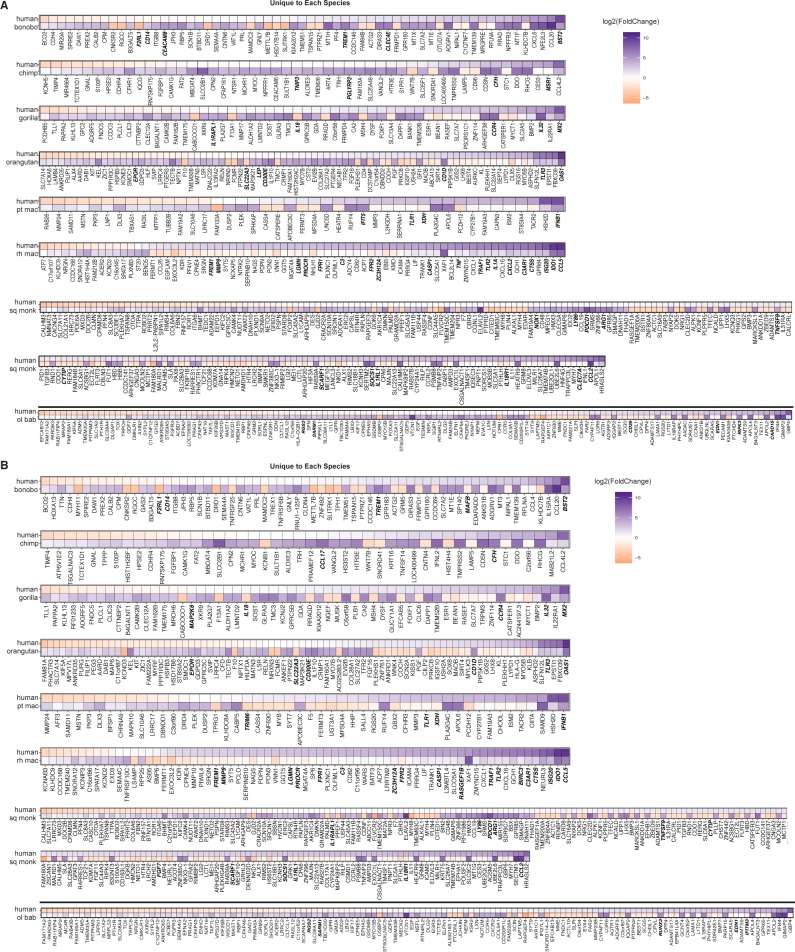
Differential expression profiles on a species-by-species basis for genes significantly different from human and unique to individual NHP species after poly(I:C) transfection. **(A, B)** For the genes found to be significantly differentially expressed compared with human (*P*_adj_ ≤ 0.05, absolute value [log_2_(foldchange)] ≥ 3) among individual NHP species, the DGE between poly(I:C)-transfected and mock-transfected samples was determined for each NHP species and is shown with the accompanying expression profile for the same genes in human. **(A, B)** Because read counts were mapped to either the human (A) or the species-specific genome (B) and the genes limited on a species-by-species basis to the ENSEMBL IDs that had a one-to-one human ortholog (referenced as “species orthologs” in the main text), each NHP species has a corresponding human DGE profile limited to those same genes. Thus, the human profiles were generated from the same set of biological samples just limited to different lists of genes for each species. For presentation purposes of the multi-species groups, the human values shown are from the olive baboon-ortholog-limited expression profile. Because these genes were found across multiple species, the expression pattern only marginally differs between the various accompanying human profiles. Genes that are decreased in expression upon poly(I:C) treatment relative to mock are shown in orange and those increased in purple, with the intensity of the color corresponding to the magnitude of the change (expressed as log_2_(foldchange)). The names of genes which are from the InnateDB list are shown in bold italics.

**Figure 4.**
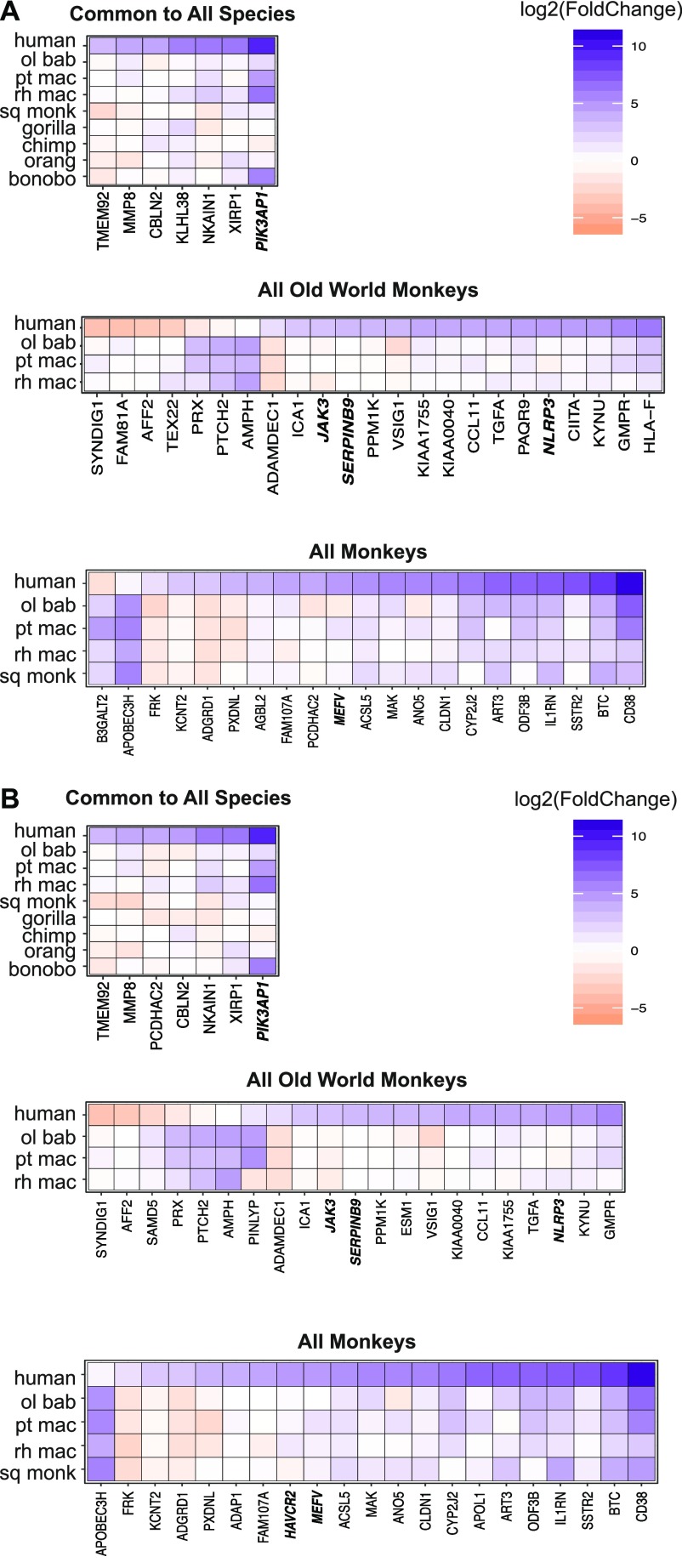
Differential expression profiles on a species-by-species basis for genes significantly different from human in subgroups of NHP species after poly(I:C) transfection. **(A, B)** For the genes found to be significantly differentially expressed compared with humans (*P*_adj_ ≤ 0.05, |log_2_(fold change)| ≥ 3) among all Old World monkeys, all monkeys, or all species (as shown in Fig 3), the DGE between poly(I:C)-transfected and mock-transfected samples was determined for each NHP species and is shown with the accompanying expression profile for the same genes in human. Because read counts were mapped to either the human (A) or the species-specific genome (B) and the genes limited on a species-by-species basis to the Ensembl IDs that had a one-to-one human ortholog (referenced as “species orthologs” in the main text), each NHP species has a corresponding human DGE profile limited to those same genes. Thus, the human profiles were generated from the same set of biological samples just limited to different lists of genes for each species. For presentation purposes of the multispecies groups, the human values shown are from the olive baboon ortholog–limited expression profile. Because these genes were found across multiple species, the expression pattern only marginally differs between the various accompanying human profiles. Genes that are decreased in expression upon poly(I:C) treatment relative to mock are shown in orange and those increased in purple, with the intensity of the color corresponding to the magnitude of the change (expressed as log_2_(fold change)). The names of genes which are from the InnateDB list are shown in bold italics.

For all these genes differentially expressed compared with human in individual NHP species or groups of species, we then examined the DGE profiles we had generated of poly(I:C) versus mock-transfected cells for each species (Supplemental Data 3 and 4). Thus, we aimed to find the basis for each gene’s significantly different expression compared with human. For example, genes with a negative log_2_(fold change) in comparing humans versus NHPs could be because the gene’s expression was unchanged by poly(I:C) transfection in NHPs but increased in humans, was decreased upon poly(I:C)-transfection in NHPs but less so or not at all in human, or did increase in NHPs after poly(I:C) transfection but at a magnitude lower than that for human. We prepared heat maps to answer this question for the genes “unique” to a given species (Fig S10) and then the three groups of interest (Old World monkeys, all monkeys, and all species) after mapping by the “human method” (Fig 4A) or the “species method” (Fig 4B). For these three groupings, regardless of mapping method, most genes that strongly increased upon poly(I:C) transfection in humans showed weak changes that were often nonsignificant in the NHP species for that group (Supplemental Data 6). With a few exceptions, when a gene’s expression was significantly different between treatment conditions in the NHP species, the expression of the NHP genes tended to follow the same trend as humans but at a weaker magnitude. In the smaller group of genes different across all species compared with human, all the genes except one demonstrated decreased or negligible change in expression after poly(I:C) transfection in the NHP species but increased expression in humans. The exception, an InnateDB gene phosphoinositide-3-kinase adaptor protein 1 (*PIK3AP1*; plays a role in immune cell development and controlling cytokine production), stood out as being up-regulated, albeit at a lower magnitude than in humans, in squirrel monkey, gorilla, and bonobo but negligibly changed in the remaining species. Between mapping methods for these smaller subgroups, most genes overlapped.

Supplemental Data 6. These files give the log_2_(fold change) values (poly(I:C)–transfected versus mock-transfected) for the genes either uniquely differentially expressed compared with humans upon poly(I:C) treatment in a single NHP species or in a group of NHP species (i.e., Old World Monkeys). Please see the README file for this dataset for further details.

For the genes “unique” to each of the great ape species, most were significantly different upon poly(I:C) transfection, whereas for these same genes in humans, the change was negligible (Fig S10 and Supplemental Data 6). The number of nondifferentially expressed genes was more evenly split between human and each of the macaque species (Fig S10 and Supplemental Data 6). In accordance with the summary data in Fig 3, most genes in the rhesus samples had lower log_2_(fold change) values compared with humans, with up-regulation occurring in humans but nonsignificant or low-level increases in rhesus. The higher number of genes for squirrel monkey and olive baboon is likely reflective of their more distant relationship to human (Fig S10 and Supplemental Data 6). For both of these NHP species, there was a subgroup of genes whose expression was clearly up- or down-regulated after poly(I:C) treatment that were nonsignificantly or negligibly different in the human samples. Likewise, the opposite trend was seen at either extreme of the human genes, with squirrel monkey and, to a lesser extent olive baboon, showing smaller or nonsignificant changes in genes that were strongly up- or down-regulated in human.

### Enrichment for cell-intrinsic immune response pathways conserved across all species

**Figure 5.**
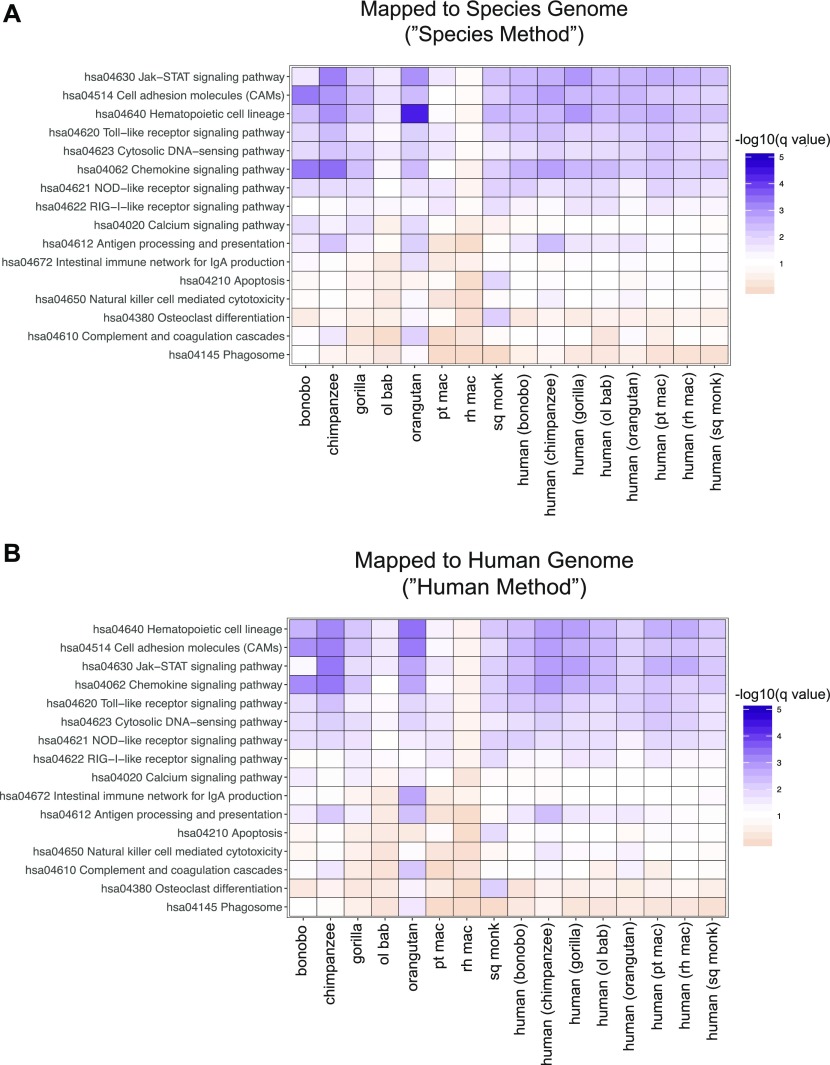
Enrichment of genes involved with cell-intrinsic immune pathways dominate the response to poly(I:C) across all species. **(A, B)** Read counts mapped to the human (A) or species-specific genomes (B) were limited on a species-by-species basis to the Ensembl IDs that had a one-to-one human ortholog (referenced as “species orthologs” in the main text). The resultant genes were then analyzed using GAGE, with the only genes excluded being those that lacked an Entrez ID (the IDs used by GAGE) and/or had a missing *P*_adj_ value. A heat map is shown of pathways (the row labels) with a *q* value ≤ 0.09 for at least one species (the column labels), with the cells colored by −log_10_(*q* value) so that cells meeting the cutoff are shades of purple and those that do not are shades of orange, with white set at the cutoff of −log_10_(0.09). Because genes were limited on a species-by-species basis to those that had a one-to-one human ortholog, each NHP species has a human profile that was limited to that same gene set (the eight right most columns of each heat map). The reads were derived from the same experimental samples—only the downstream analysis differs in terms of the genes that were included by their ortholog status. Source data are available for this figure.

Biological outcomes are not necessarily dictated by individual genes but rather the concerted efforts of multiple genes acting in tandem. Although innate immune genes were present amongst genes differentially expressed in NHP but not in human samples, it was unclear if this handful of players was resulting in large-scale differences in the cell-intrinsic immune responses of these various species to poly(I:C). Furthermore, as only one time point post-transfection was tested in this study, it is impossible to determine if genes with altered expression from humans are differentially stimulated by poly(I:C) or the result of varying transcriptional kinetics. For a broader overview of these DGE profiles, we performed gene set analysis, whereby data are assessed for an enrichment of genes related in some way, such as their participation in the same pathway or biological function (36, 37, 38, 39). Because each species was limited to its own number of one-to-one orthologs, we prepared a human DGE profile for each species limited in the same way to ease comparisons. We used the R package Generally Applicable Gene-set Enrichment (GAGE) (40) to determine the Kyoto Encyclopedia of Genes and Genomes (KEGG) (41, 42, 43) pathways with the most significantly altered gene expression after poly(I:C) transfection. To reflect the nature of pathways in biological systems, bidirectional changes in gene expression were considered when determining pathway enrichment. For each NHP species besides rhesus macaque and regardless of the mapping method, genes were significantly enriched (q_val_ ≤ 0.09) for multiple pathways known to be important for cell-intrinsic immune responses, either directly or indirectly, including Toll-like receptor (TLR) signaling, nucleotide-binding oligomerization domain (NOD)-like receptor signaling, cytosolic DNA sensing, JAK-STAT signaling, antigen processing and presentation, and chemokine signaling (Fig 5; the latter two were also significant for mice, Table 5). Although all the NHPs had pathway profiles that corresponded well with the accompanying human profile, rhesus macaque failed to meet the significance cutoff for any pathway. This was not due to limiting the list of genes to one-to-one orthologs as evidenced by the accompanying human profile, which was limited to the same gene list. The top-ranking pathways were the same as the other species but likely did not pass the significance threshold because of the lower number of differentially expressed genes relative to the other species. In some instances, such as NOD-like receptor signaling for olive baboon and retinoic acid-inducible gene I–like receptor signaling for bonobo, the significance value fell just outside of the cutoff, indicating that although perhaps not to the same extent as the other NHPs, these pathways are still important. Apoptosis and osteoclast differentiation genes stood out as clearly significant in their enrichment only for squirrel monkey and some of the corresponding human profiles. Although apoptosis is less unexpected, osteoclast differentiation could be appearing as a result of the common origin of osteoclasts and immune cells from hematopoietic cells and the role of TLR stimulation in disrupting osteoclast differentiation (44).

**Table 5.** Top 12 pathways for which genes are enriched in mice after poly(I:C) transfection.

Kyoto Encyclopedia of Genes and Genomes pathway	*q* value
mmu04514 cell adhesion molecules	0.005223675
mmu04612 antigen processing and presentation	0.076815675
mmu04062 chemokine signaling pathway	0.076815675
mmu04620 TLR signaling pathway	0.101942397
mmu04672 intestinal immune network for IgA production	0.112781502
mmu04623 cytosolic DNA-sensing pathway	0.112781502
mmu04621 NOD-like receptor signaling pathway	0.112781502
mmu04145 phagosome	0.112781502
mmu04610 complement and coagulation cascades	0.232711025
mmu04640 hematopoietic cell lineage	0.235522039
mmu02010 ABC transporters	0.235522039
mmu04622 RIG-I-like receptor signaling pathway	0.252956436

Together, these data mimicking a viral infection serve as a first step in comparing the cell-intrinsic immune responses of the most diverse panel of NHP species to date. In using a synthetic compound as a proxy for RNA virus infection, we were able to analyze transcriptomic responses without having to account for species-specific permissiveness and/or susceptibility to a pathogen. While there were of course differences in individual gene expression profiles after poly(I:C) transfection across species, these ultimately resulted in similar outcomes, activating pathways important to cell-intrinsic immunity. We compared our findings to a recent study of rhesus, mouse, and human primary DFs transfected with poly(I:C) for 4 h (26). Although our time point was 20 h later, some of these early genes (filtered for primates to those with a one-to-one human ortholog) remained up-regulated in these same species at 24 h (*P*_adj_ ≤ 0.05, log_2_(foldchange) ≥ 3) − ∼22% for rhesus, ∼38% for mouse, and ∼60% for human (Supplemental Data 7). This comparison highlights the rapid nature of cell-intrinsic immune responses and the sustained transcription of these responses promoted by continued ISG production. However, it also suggests species–species differences in the kinetics of such responses that will be an important area for further research. Similarly, among the 62 genes that were commonly increased in response to type I IFN treatment in species far more divergent from the ones we studied (bats, chicken, horse, sheep, rat, cow, human, and pig) (27), from 50% (rhesus) to 77% (squirrel monkey) of the genes that had a one-to-one human ortholog in each of our NHP species (>90% of the 62 genes) were also up-regulated 24 h post-poly(I:C) treatment using the same cutoffs as those of Shaw et al (*P*_adj_ ≤ 0.05, log_2_(foldchange) ≥ 2; Supplemental Data 8). In comparison, our human samples showed ∼80%.

Supplemental Data 7. These files contain the DGE profiles from our experiments for the genes found to be differentially expressed in mouse, rhesus and human by Hagai et al. in their 2018 work (26).

Supplemental Data 8. These files contain the DGE profiles from our experiments for the genes identified as “Core ISGs” in the work of Shaw et al in 2017 (27).

Although we observed a high level of similarity across these NHP species, this is not to say that in the context of a viral infection, all species would mount the same response. In transfecting the cells with poly(I:C), we provoked a response that is not tempered by the antagonism of viral proteins and thus closer to what the potential cell-intrinsic immune response of these cells would be if left uninhibited by the actions of viral proteins. As a result, the data presented here do not presume to predict the susceptibility of these species to particular viral pathogens or whether other cell types from these species would react the same way. We tested the assumption that the species’ response to poly(I:C) would be highly similar as this had not previously been formally demonstrated for all of these species. Thus, although striking differences between species would have been of great interest, a quantitative demonstration of their similarity is still very useful. Knowing the unfettered capabilities of these cells in terms of cell-intrinsic immunity provides a solid foundation to explore how this more maximal response is potentially dampened by the interplay of host and viral proteins.

In addition, as alluded to above, because we only examined one time point, the kinetics of the transcriptomic response provides another area for future analysis that could demonstrate interesting differences across species. These data will serve as an important baseline for such work, showing what transcriptional responses are possible and how they are impacted upon exposure to a pathogen. Furthermore, because the current study only examined poly-A–containing RNA transcripts, we cannot exclude the role of other RNA species, such as long non-coding RNAs that are becoming of increasing interest in NHP genomics ((45), reviewed in reference 46).

**Figure S11.**
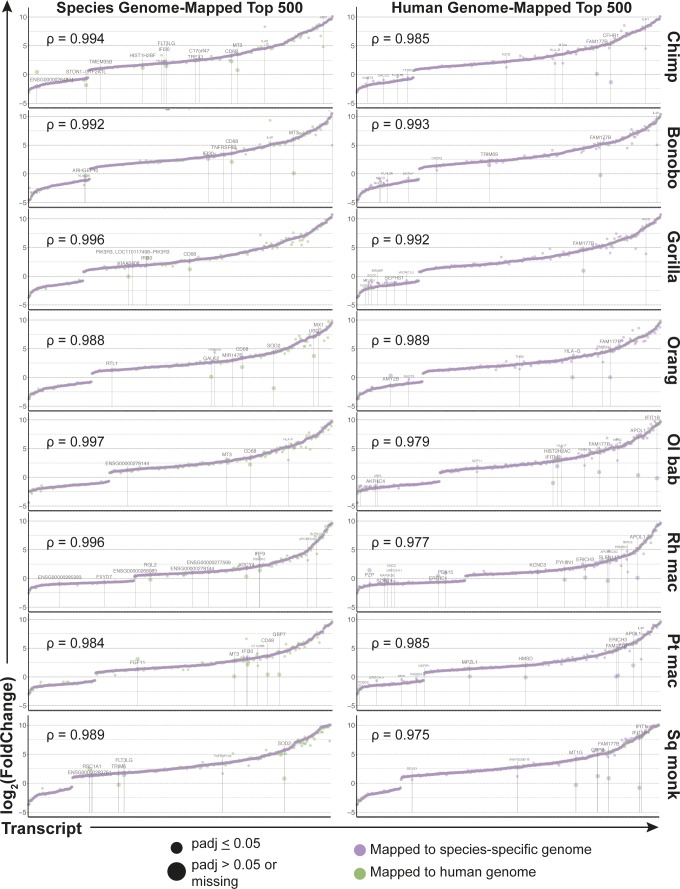
Comparing the top 500 most significantly differentially expressed genes between the two mapping methods. In the left column are the top 500 most significantly (based off *P*_adj_ value) differentially expressed genes found after using the species-specific genome for mapping, with the genes arranged by increasing log_2_(foldchange) for the species mapping method (shown in purple). The corresponding log_2_(foldchange) for the same genes after using the human genome as the mapping reference are shown in green. The right column shows the inverse, with the coloring maintained, where the top 500 most significantly differentially expressed genes after mapping to the human genome are shown by increasing log_2_(foldchange). Each row is a different species and the Spearman coefficient of the log_2_(foldchange) values (after omitting any missing values of which there were no more than five) in each plot is shown. Data information: genes are labeled with their gene symbol if any of the following criteria were met: (i) the ratio of the log_2_(foldchange) from each method for a given gene was less than 0.67 or greater than 1.5 AND the absolute value of the log_2_(foldchange) by at least one of the mapping methods was ≥2, (ii) the log_2_(foldchange) was determined as “NA” by the mapping method not used to create the list of 500 most significant genes, and (iii) the *P*_adj_ value was missing or nonsignificant. For genes in the final category, the point and label are shown enlarged. Where no gene symbol was listed, the human ENSEMBL ID was used.

Finally, our parallel workflow allowed us to compare the benefits of mapping to the human versus the species-specific genome for these NHP species. The broader outcomes, such as the pathway enrichment analysis, were strongly similar between the two methods, and as noted earlier, the additional alignment gained by using species-specific genomes was often to regions with no features yet assigned. As a further comparison of the two mapping methods, we examined the top 500 most significantly differentially expressed genes ultimately identified after either the “species method” or the “human method.” Depending on the species, 88–93% of the top 500 most significant genes overlapped between the two mapping methods (Fig S11 and Supplemental Data 9). For each set of 500 genes from a given mapping method, we compared the log_2_(fold change) and significance for those same genes from the alternative mapping method. The log_2_(fold change) values were highly correlated and we observed for each species less than 15 genes that were (i) significantly differentially expressed by one approach (*P*_adj_ ≥ 0.05) but not the other or (ii) had a log_2_(fold change) value that was 1.5 times higher or lower in one mapping method versus the other and also had a |log_2_(fold change)| ≥ 2 in at least one of the mapping methods. For genes that met these criteria, the differing expression between mapping methods was due to a variety of reasons. Reads which mapped to a region that had overlapping annotations in one of the reference genomes but not the other caused those reads to be excluded for not mapping to a unique feature. In other instances, multiple transcripts of the same gene were annotated covering a large region in one reference but not the other, so although alignment was observed by either mapping reference, an annotation only existed for one of the genomes.

Supplemental Data 9. These are the log_2_(fold change) and *P*_adj_ values for the genes in NHP species that were found to be in the top 500 most significantly differentially expressed genes after using the human genome mapping approach (file name includes “HumanTop500Sig”) versus those after using the species-specific genome mapping approach (file name includes “SpeciesTop500Sig”). In each file, the *P*_adj_ and log_2_(fold change) values are shown for the same genes but by the other mapping approach. These are the data that were used to generate Fig S11.

These findings are in line with the general observation that the human genome is more well annotated than those of NHPs and underscores the importance of improving the feature assignment of these sequences. RNA-Seq datasets such as ours can help identify novel exons or in some cases aid in rectifying feature assignments at regions where exons were incorrectly annotated. For example, as alluded to above, a given gene in the human genome may have more annotated transcript variants that can sometimes cover a larger region and lead to read assignment, whereas the orthologous NHP gene, with sometimes only one listed variant, has no reads assigned to it as the reads map but to an area outside of the limited region where the single variant was annotated. In making these data available, we anticipate that they will facilitate continued efforts to annotate the genomes of NHP species and to identify additional transcript variants of already annotated genes as well as completely novel transcripts unobserved in humans. As with the “1,000 Genomes Project” for human samples (47), having more sequencing data available from multiple individuals for a given species aids in identifying single-nucleotide polymorphisms and other sources of genomic variation to improve annotations. We explicitly strove to include at least one male and one female with varying ages across the three donors. This is far more reflective of the natural variance that exists in any outbred species population and makes our sequencing reads all the more useful, especially for genomes, such as that of orangutan, sourced from a single individual. We hope our present analysis and this data collection more broadly will serve as a springboard for additional evolutionary analysis and comparisons.

## Materials and Methods

### DFs

Mouse DFs from C57BL/6 mice were purchased from Cell Biologics. Primary human DFs were obtained from American Type Culture Collection (PCS-201-012). All the NHP DFs were purchased from the Coriell Institute for Medical Research with the exception of two squirrel monkey donors (SQMA and SQMB), which were derived from skin biopsies generously provided by Robert Lanford (Southwest Biomedical Research Center). For any of the DF donor species that are on the United States Fish and Wildlife Services endangered or threatened species list, the Coriell Institute has the appropriate and required documentation of breeding records (Supplemental File 1) indicating captivity before or birth after November 18, 1976. The squirrel monkey skin biopsies were obtained after terminal necropsy using Princeton University Institutional Animal Care and Use Committee–approved protocols (#1930) and shipped overnight on wet ice to Princeton University. Upon arrival, the skin was prepped and DFs isolated according to previously published protocols (48). In brief, the skin biopsies were scraped to remove connective tissue, cut into smaller pieces, and digested overnight at 4°C in HBSS without Ca^2+^ and Mg^2+^ (Thermo Fisher Scientific), containing 1 ml dispase (5,000 caseinolytic units/ml; Corning) for every 9 ml of HBSS containing final concentrations of 100 mg/ml streptomycin, 100 U/ml penicillin, and 250 ng/ml amphotericin B (HyClone). After digestion, the epidermis was removed and discarded, whereas the remaining dermis was cut into smaller pieces less than a few square millimeter in area. These pieces were moistened with DMEM and pressed into a six-well plate scored with a razor blade. The dermis was maintained in DMEM containing 10% FBS and 1% vol/vol penicillin/streptomycin solution at 37°C, 5% CO_2_. Media was changed every 4–5 d and fibroblast growth was typically observed within 1 wk of culture. Once sufficient outgrowth had occurred, the dermis was removed from the plate and the fibroblasts expanded into larger cultures. Complete donor information as provided by these sources can be found in Table 4.

Supplemental File 1. The breeding records and documentation for DFs acquired from species on the United States Fish and Wildlife Services endangered or threatened species list.

### Cell culture

All cells, unless otherwise stated, were grown under standard conditions in DMEM (Thermo Fisher Scientific) containing 10% FBS and 1% vol/vol penicillin/streptomycin. Upon reaching confluency, the cells were trypsinized with 0.05% trypsin/EDTA and split 1:3.

### polyI:C transfections

DFs were transfected with ∼0.05 μg of high molecular weight poly(I:C) (Invivogen) per square centimeter (0.5 μg/well in a six-well format in triplicate for samples undergoing RNA-Seq and 0.1 μg/well in duplicate in a 24-well format for relative RT-qPCR validation experiments) using X-tremeGENE HP DNA Transfection Reagent (Roche) 1 μl per μg of poly(I:C) in Opti-MEM (Thermo Fisher Scientific). Mock transfections were performed in parallel under the same conditions minus poly(I:C). Collected cell lysates (350 μl volume) were immediately frozen at −80°C until RNA extraction performed.

### RNA extraction, cDNA library preparation, and RNA sequencing

Total RNA was isolated from DFs using the RNeasy Mini Kit (QIAGEN) according to the manufacturer’s instructions. For all samples undergoing RNA-Seq, the quality and concentration of RNA was assessed on an Agilent 2100 Bioanalyzer (Agilent Technologies). All samples subsequently sequenced had an RNA Integrity Number of ≥8. The poly-A–containing RNA transcripts in the total RNA samples were converted to cDNA and amplified after the Smart-seq2 method (49). Sequencing libraries were made from the amplified cDNA samples using the Nextera kit (Illumina), assigning a unique barcode to each of the libraries to be sequenced together. The cDNA samples and libraries were examined on the Bioanalyzer (Agilent) DNA HS chips for size distribution and quantified by Qubit fluorometer (Invitrogen). The RNA-Seq libraries were pooled together in equal amounts and sequenced on the Illumina HiSeq 2500 Rapid Flowcell as single-end 75-nt reads following the standard protocol, giving a range of 15–20 million reads per sample. Raw sequencing reads were filtered by Illumina HiSeq Control Software and only pass-filter reads were used for further analysis.

### RT-qPCR of select ISGs

RT-qPCR of total RNA isolated from the 24-well format poly(I:C) transfections was performed using the Luna Universal One-Step RT-qPCR kit (New England BioLabs, Inc.) according to the manufacturer’s directions. Primer sequences can be found in Table 6. In brief, a master mix was prepared that comprised 10 μl of 2× Luna Universal One-Step Reaction Mix (2×), 1 μl of 20× Luna WarmStart RT Enzyme Mix, 0.8 μl of a 10 μM stock of each primer, and 5.4 μl of nuclease-free water per reaction. Each well received 18 μl of the appropriate master mix and 2 μl of the RNA being assayed. The following PCR program was then run on an Applied Biosystems Step One Plus qPCR machine (Life Technologies): denatured at 55°C for 10 min, 95°C for 1 min, followed by 40 cycles of 95°C for 10 s and 60°C for 60 s. Last, a melt curve was performed at 95°C for 10 s, 65°C for 10 s, 95°C for 10 s, and 50°C for 5 s.

**Table 6.** Primers for RT-qPCR of IFNβ, MX1, OASL, and HPRT1 for the species used in this study.

Species	Mx1	OasL (Oasl1 for mouse)	HPRT1	IFNB
Rhesus	PU-O-2198, -2532	PU-O-4828, -4829	PU-O-2409, -1469	PU-O-2211, -2215
Bonobo	PU-O-2195, -2196	PU-O-2206, -2209	PU-O-1468, -1469	PU-O-2211, -2212
Chimpanzee	PU-O-2195, -2196	PU-O-2206, -2209	PU-O-1468, -1469	PU-O-2211, -2212
Pigtailed macaque	PU-O-2198, -2196	PU-O-4828, -4829	PU-O-1468, -1469	PU-O-2211, -2215
Squirrel monkey	PU-O-2525, -2526	PU-O-2529, -2530	PU-O-1468, -1469	PU-O-2475, -2476
Orangutan	PU-O-2197, -2199	PU-O-2206, -2208	PU-O-1468, -1469	PU-O-2211, -2214
Mouse	PU-O-4236, -4237	PU-O-4856, -4857	PU-O-4212, -4213	PU-O-4240, -4241
Olive baboon	PU-O-2196, -2198	PU-O-2207, -2210	PU-O-1468, -1469	PU-O-2211, -2215
Gorilla	PU-O-2195, -2196	PU-O-2206, -2208	PU-O-1468, -1469	PU-O-2211, -2216
Human	PU-O-2195, -2196	PU-O-2206, -2208	PU-O-1468, -1469	PU-O-2211, -2212
Forward primers (5′ to 3′)			Reverse primers (5′ to 3′)	
PU-O-1468	CCTGGCGTCGTGATTAGTGAT		PU-O-1469	AGACGTTCAGTCCTGTCCATAA
PU-O-2195	GTTTCCGAAGTGGACATCGCA		PU-O-2196	CTGCACAGGTTGTTCTCAGC
PU-O-2197	CTTTCCGAAGTGGACATCGCA		PU-O-2199	CTGCACAGATTGTTCTCAGC
PU-O-2198	CTTTCTGAAGTGGACATTGTA		PU-O-2208	CACAGCGTCTAGCACCTCTT
PU-O-2206	CTGATGCAGGAACTGTATAGC		PU-O-2209	CACAGTGTCTAGCACCTCTT
PU-O-2207	CTGATGCAGGAACTGTACAGC		PU-O-2210	CACAGCATCTAGAACCTCCT
PU-O-2211	GCTTGGATTCCTACAAAGAAGCA		PU-O-2212	ATAGATGGTCAATGCGGCGTC
PU-O-2409	GATTAGTGATGATGAACCA		PU-O-2214	GTAGATGGTCAATGCCGCGTC
PU-O-2475	ACTTGGATTCCTACAAAGAAGAA		PU-O-2215	ATAGATGGTCAATGCAGCGTC
PU-O-2525	CTTTCCGAAGTGGGAGTCGGA		PU-O-2216	ATAGATGGTCAATGCCGCGTC
PU-O-2529	CTGACACAGGAGCTGTATGCC		PU-O-2476	ATAGACGATTAATGCCACGTC
PU-O-4212	TCAGTCAACGGGGGACATAAA		PU-O-2526	CTGTACAGGTTGTTCTCGGC
PU-O-4236	GGTCTTGGATGTGATGCGGA		PU-O-2530	CACAGTGTCCAGCACCTCTT
PU-O-4240	TGTCCTCAACTGCTCTCCAC		PU-O-2532	TGCACAGGTTGTTCTCAGC
PU-O-4828	CCATCGTGCCTGCCTACAGAG		PU-O-4213	GGGGCTGTACTGCTTAACCAG
PU-O-4856	CAGGAGCTGTACGGCTTCC		PU-O-4237	TGCTGACCTCTGCACTTGAC
			PU-O-4241	ACCACCACTCATTCTGAGGC
			PU-O-4829	CTTCAGCTTAGTTGGCCGATG
			PU-O-4857	CCTACCTTGAGTACCTTGAGCAC

### RNA-Seq analysis

Using the Galaxy system (50) provided by Princeton University, short reads were aligned to the human or the originating species’ genomes (see Table 2) using RNA STAR (51, 52) (Galaxy version 2.6.0b-1) with default parameters. Counts were generated using featureCounts with default settings (53) (Galaxy version 1.6.3+galaxy2), downloaded, and read into R (54) version 3.5.2 (December 20, 2018) using scripts run in RStudio (55) version 1.1.463. DGE was determined using DESeq2 (56) (version 1.22.2) using the standard filters and nbinomWaldTest (57). The design used to model the samples was ∼species + species:donor.n + treatment + species:treatment with various contrasts set as shown in the R code and as described in the README files for the Datasets EV3–5. Transcript counts were normalized using DESeq2 default options and transformed using the regularized log_2_ function in DESeq2 before PCA plotting. Results were extracted from the DESeq2 analysis and annotated using Bioconductor’s AnnotationDbi (version 1.44.0) org.Hs.eg.db and org.Mm.eg.db. For further details concerning the R packages used and the specific conditions used for analysis, R code can be accessed at https://github.com/aploss/polyIC-dermal-fibroblasts-RNA-Seq.

### Statistical analysis

Statistical analysis for RT-qPCR data was performed with GraphPad Prism software as indicated in the figure legends.

### Data access

The RNA-Seq data generated in this study are deposited in the National Center for Biotechnology Information Gene Expression Omnibus database (accession number GSE105160).

## Supplementary Material

Reviewer comments
