# Supplementary material for: Conservation of cell-intrinsic immune responses in diverse nonhuman primate species
Source: Life Sci Alliance. 2019 Oct 24;2(5):e201900495. doi: 10.26508/lsa.201900495 (PMC6814850; doi:10.26508/lsa.201900495)
Supplement: Supplementary file 1 [file LSA-2019-00495_SdataF1.pdf]

PR00051

Print Pedigree Report

Specimen Report

November 15, 2002 10:06:27 AM

[illegible] Species Information System

Specimen SD-WAP/694047

Names:

Taxonomic:	Common:	Family:	Order:
Pan paniscus	Pygmy chimpanzee	Hominidae	Primates

Birth Information:

Sex:	Birth Location:	Birth date:	Birth type:	Hybrid:	Rearing:
Female	SD-WAP	8 Feb 1994	Captive born		

Visits:

Date:	Aquisition:	Vendor/LocalID:	Holder:	Disposition:	Recipient/LocalID:	Date:
8 Feb 1994	Birth		SD-WAP/694047			

Identification:

Date:	Type:	Identifier:	Location:	Comments:
8 Feb 1994	House Name	MALELA		at SD-WAP
7 Oct 1998	Transponder ID	00012183DB		at SD-WAP

Sex Information:

Date:	Sex:	Comments:
8 Feb 1994	Female	at SD-WAP

Parents:

Date:	Parent type:	ID:	Location:	Comments:
8 Feb 1994	Sire	181056	SD-WAP	while at SD-WAP
8 Feb 1994	Dam	282014	SD-WAP	while at SD-WAP

Specimen Report

SIRE

ISIS GAN: MIG12-29876876 181056

Pan paniscus Bonobo Pan paniscus

Order: Primates **Family:** Hominidae

Start Date: Jan 01, 1800 **End Date:** Sep 14, 2012

Basic Animal Information

**Sex - Contraception:**	Male -	**Status:**	Alive
**Birthdate - Age:**	Jul 02, 1980 - 32Y,2M,15D	**Preferred ID:**	181056
**Origin:**	Yerkes National Primate Research Ctr	**Rearing:**	Undetermined
**Birth Type:**	Captive Born	**Hybrid Status:**	Not a hybrid
**Sire:**	MULTIPLE	**Dam:**	MIG12-25333283 | YERKES P1
**Current Collection:**	Main Institution Animal Collection	**Collection Trip:**	
**Clutch / Litter:**			

Physical History:

Reported By	Date	Source / ID	Holder / ID	Term Type	Recipient / ID
YERKES	Jul 02, 1980		YERKES / P6	Birth/Hatch (Physical and Ownership)	
SANDIEGOZ	Mar 26, 1981	YERKES / P6	SANDIEGOZ / 181056	Trade From(Physical and Ownership)	
YERKES	Mar 26, 1981		YERKES / P6	Donation To (Physical and Ownership)	SANDIEGOZ / 181056
SANDIEGOZ	Jul 11, 1989		SANDIEGOZ / 181056	Undetermined (Physical and Ownership)	SD-WAP / 181056
SD-WAP	Jul 11, 1989	SANDIEGOZ / 181056	SD-WAP / 181056	Undetermined (Physical and Ownership)	
JACKSONVL	Oct 23, 2008		JACKSONVL / 808387	Loan In From (Physical Only)	
SD-WAP	Oct 23, 2008		SD-WAP / 181056	Loan Out To (Initial Transaction - Physical Only)	JACKSONVL / 808387

Ownership History:

Reported By	Date	Source / ID	Owner / ID	Term Type	Recipient / ID
YERKES	Jul 02, 1980		YERKES / P6	Birth/Hatch (Physical and Ownership)	
SANDIEGOZ	Mar 26, 1981	YERKES / P6	SANDIEGOZ / 181056	Trade From(Physical and Ownership)	
YERKES	Mar 26, 1981		YERKES / P6	Donation To (Physical and Ownership)	SANDIEGOZ / 181056
SANDIEGOZ	Jul 11, 1989		SANDIEGOZ / 181056	Undetermined (Physical and Ownership)	SD-WAP / 181056
SD-WAP	Jul 11, 1989	SANDIEGOZ / 181056	SD-WAP / 181056	Undetermined (Physical and Ownership)	
JACKSONVL	Oct 23, 2008		SD-WAP / 181056	Reported Owner	
SD-WAP	Oct 23, 2008		SD-WAP / 181056	Reported Owner	

Reported By	Date	Type	Identifier	Location	Comments
JACKSONVL	Oct 23, 2008	Local ID	808387		
JACKSONVL	Oct 07, 1998	Transponder	00-0121-29FF		Legacy SLocation:<br>Legacy Comment: Trovan
SD-WAP	Oct 07, 1998	Transponder	00012129FF		
JACKSONVL	Mar 26, 1981	ID elsewhere	181056		Legacy SLocation: SANDIEGOZ<br>Legacy Comment: at San Diego Zoo
SANDIEGOZ	Mar 26, 1981	Tattoo	SB94		
SANDIEGOZ	Mar 26, 1981	House Name	AKILI		
JACKSONVL	Mar 26, 1981	Tattoo	SB94		
JACKSONVL	Jul 02, 1980	Studbook Name	YERK 1		
YERKES	Jul 02, 1980	House Name	AKILI		
JACKSONVL	Jul 02, 1980	House Name	AKILI		
YERKES	Jul 02, 1980	Intl Stdbk#	94		
JACKSONVL	Jul 02, 1980	Intl Stdbk#	0094		
JACKSONVL	Jul 02, 1980	ID elsewhere	P6		Legacy SLocation: YERKES<br>Legacy Comment: at Yerkes Primate Center
YERKES	Jul 02, 1980	Studbook Name	YERK 1		
YERKES		Local ID	P6		
SD-WAP		Local ID	181056		
SANDIEGOZ		Local ID	181056		

Sex Information:

Reported By	Date	Sex	Comments
JACKSONVL	Oct 23, 2008	Male	
YERKES	Jul 02, 1980	Male	

Parent Info:

In ZIMS	Parent Info	Type / Probability	Birth Date	Reported By
True	MIG12-25333283	Dam/100%	Mar 26, 1970	JACKSONVL
True	MIG12-25333283	Dam/100%	Mar 26, 1970	YERKES
True	4893096	Sire/100%	Jan 01, 1971	YERKES
False	P4/YERKES	Sire/100%		JACKSONVL

Specimen Report

FATHER OF SIRE

ISIS GAN: 4893096

Pan paniscus — Bonobo

Order: Primates — **Family:** Hominidae

Start Date: Jan 01, 1800 — **End Date:** Sep 14, 2012

Basic Animal Information

**Sex - Contraception:**	Male -	**Status:**	Dead
**Birthdate - Age:**	Jan 01, 1971 - 34Y,6M,21D	**Preferred ID:**	
**Origin:**	Congo, The Democratic Republic Of The -	**Rearing:**	Parent
**Birth Type:**	Wild Born	**Hybrid Status:**	Not a hybrid
**Sire:**	| JACKSONVL WILD	**Dam:**	| JACKSONVL WILD
**Current Collection:**		**Collection Trip:**	
**Clutch / Litter:**			

Physical History:

Reported By	Date	Source / ID	Holder / ID	Term Type	Recipient / ID
YERKES	Mar 27, 1975	KINSHASA / UNK	YERKES / P4	Donation From (Physical and Ownership)	
YERKES	Apr 15, 1998		YERKES / P4	Loan Out To (Initial Transaction - Physical Only)	JACKSONVL / UNK
JACKSONVL	Apr 15, 1998		JACKSONVL / 898051	Loan In From (Physical Only)	
JACKSONVL	Jul 22, 2005		JACKSONVL / 898051	Death (Physical and Ownership)	

Ownership History:

Reported By	Date	Source / ID	Owner / ID	Term Type	Recipient / ID
YERKES	Mar 27, 1975	KINSHASA / UNK	YERKES / P4	Donation From (Physical and Ownership)	
YERKES	Apr 15, 1998		YERKES / P4	Reported Owner	
JACKSONVL	Apr 15, 1998		YERKES / P4	Reported Owner	
YERKES	Jul 27, 1999		YERKES / P4	Donation To (Ownership Only)	JACKSONVL /
JACKSONVL	Sep 07, 1999	YERKES / P4	JACKSONVL / 898051	Donation From (Ownership Only)	
JACKSONVL	Jul 22, 2005		JACKSONVL / 898051	Death (Physical and Ownership)	

Identifiers:

Reported By	Date	Type	Identifier	Location	Comments
JACKSONVL	Apr 15, 1998	Local ID	898051		
YERKES	Mar 27, 1975	House Name	BOSONDJO		
JACKSONVL	Mar 27, 1975	House Name	BOSONDJO		
JACKSONVL	Mar 27, 1975	Intl Stdbk#	64		
YERKES	Mar 27, 1975	Intl Stdbk#	64		
YERKES		Local ID	P4		

Reported By	Date	Sex	Comments
JACKSONVL	Apr 15, 1998	Male	
YERKES	Mar 27, 1975	Male	

Parent Info:

In ZIMS	Parent Info	Type / Probability	Birth Date	Reported By
False	WILD/230900000	Dam/100%		JACKSONVL
False	WILD/230900000	Sire/100%		JACKSONVL

Death Information:

Reported By	Death Type	Date	Carcass Disposition	Necropsy Topology	Necropsy Etiological
JACKSONVL	Undetermined		Undetermined	Necropsy Planned Later	Indeterminate

Circumstance of death: Other/Unknown; Carcass disposition: Unknown; Carcass recipient: (not supplied); Necropsy - Topological: Necropsy Planned Later; Necropsy - Etiological: (not supplied)

Body Part	Institution	Recipient ID	Date Sent	Date Received	Genetic Results

Specimen Report

MOTHER OF SIRE

ISIS GAN: MIG12-25333283

Pan paniscus — Bonobo

Order: Primates — **Family:** Hominidae

Start Date: Jan 01, 1800 — **End Date:** Sep 14, 2012

Basic Animal Information

**Sex - Contraception:**	Female - Undetermined/Active	**Status:**	Alive
**Birthdate - Age:**	Mar 26, 1970 - 42Y,5M,21D +/-2Y	**Preferred ID:**	
**Origin:**	Unknown Location	**Rearing:**	Parent
**Birth Type:**	Wild Born	**Hybrid Status:**	Not a hybrid
**Sire:**	MULTIPLE	**Dam:**	MULTIPLE
**Current Collection:**		**Collection Trip:**	
**Clutch / Litter:**			

Physical History:

Reported By	Date	Source / ID	Holder / ID	Term Type	Recipient / ID
YERKES	Mar 27, 1975		YERKES / P1	Loan In From (Physical Only)	
YERKES	May 04, 2000		YERKES / P1	Donation To (Physical and Ownership)	ATLANTA / A01013
ATLANTA	May 04, 2000	YERKES / P1	ATLANTA / A01013	Donation From (Physical and Ownership)	
ATLANTA	May 04, 2000		ATLANTA / A01013	Loan Out To (Initial Transaction - Physical Only)	LCR / NONE
GATI	May 14, 2005		GATI / 9	Loan In From (Physical Only)	

Ownership History:

Reported By	Date	Source / ID	Owner / ID	Term Type	Recipient / ID
YERKES	Mar 27, 1975		KINSHASA / UNK	Reported Owner	
YERKES	Aug 21, 1991	KINSHASA /	YERKES / P1	Donation From (Ownership Only)	
YERKES	May 04, 2000		YERKES / P1	Donation To (Physical and Ownership)	ATLANTA / A01013
ATLANTA	May 04, 2000	YERKES / P1	ATLANTA / A01013	Donation From (Physical and Ownership)	
ATLANTA	May 04, 2000		ATLANTA / A01013	Reported Owner	
GATI	May 14, 2005		ATLANTA / A01013	Reported Owner	

Reported By	Date	Type	Identifier	Location	Comments
GATI	May 14, 2005	House Name	Matata		
GATI	May 09, 2005	Intl Stdbk#	62		
ATLANTA	May 04, 2000	House Name	Matata		
ATLANTA	May 04, 2000	Intl Stdbk#	62		
YERKES	Mar 27, 1975	Intl Stdbk#	62		
YERKES	Mar 27, 1975	House Name	MATATA		
ATLANTA		Local ID	A01013		
GATI		Local ID	9		
YERKES		Local ID	P1		

Sex Information:

Reported By	Date	Sex	Comments
YERKES	Mar 27, 1975	Female	

Parent Info:

In ZIMS	Parent Info	Type / Probability	Birth Date	Reported By
False	WILD/			ATLANTA
False	WILD/			YERKES
False	WILD/			GATI
False	WILD/			GATI
False	WILD/			YERKES
False	WILD/			ATLANTA

Specimen Report

ISIS GAN: 26298915 282014 DAM

Pan paniscus Bonobo Pan paniscus

Order: Primates **Family:** Hominidae

Start Date: Jan 01, 1800 **End Date:** Sep 14, 2012

Basic Animal Information

**Sex - Contraception:**	Female -	**Status:**	Alive
**Birthdate - Age:**	Feb 03, 1982 - 10Y,5M,21D	**Preferred ID:**	282014
**Origin:**	San Diego Zoo	**Rearing:**	Undetermined
**Birth Type:**	Captive Born	**Hybrid Status:**	Not a hybrid
**Sire:**	MIG12-26751591 | SANDIEGOZ 180343	**Dam:**	27751557 | SANDIEGOZ 172284
**Current Collection:**	Main Institution Animal Collection	**Collection Trip:**	
**Clutch / Litter:**			

Physical History:

Reported By	Date	Source / ID	Holder / ID	Term Type	Recipient / ID
SANDIEGOZ	Feb 03, 1982		SANDIEGOZ / 282014	Birth/Hatch (Physical and Ownership)	
SANDIEGOZ	Jun 20, 1988		SANDIEGOZ / 282014	Loan Out To (Initial Transaction - Physical Only)	CINCINNAT / M5062
CINCINNAT	Jun 21, 1988		CINCINNAT / M5062	Loan In From (Physical Only)	
CINCINNAT	Jul 02, 1992		CINCINNAT / M5062	Loan Return to Owner (Physical Only)	SANDIEGOZ / 282014
SANDIEGOZ	Jul 02, 1992	CINCINNAT / M5062	SANDIEGOZ / 282014	Loan Return to Us (Physical Only)	
SANDIEGOZ	Jul 24, 1992		SANDIEGOZ / 282014	Undetermined (Physical and Ownership)	SD-WAP / 282014
SD-WAP	Jul 24, 1992	SANDIEGOZ / 282014	SD-WAP / 282014	Undetermined (Physical and Ownership)	

Ownership History:

Reported By	Date	Source / ID	Owner / ID	Term Type	Recipient / ID
SANDIEGOZ	Feb 03, 1982		SANDIEGOZ / 282014	Birth/Hatch (Physical and Ownership)	
SANDIEGOZ	Jun 20, 1988		SANDIEGOZ / 282014	Reported Owner	
CINCINNAT	Jun 21, 1988		SANDIEGOZ / 282014	Reported Owner	
SANDIEGOZ	Jul 24, 1992		SANDIEGOZ / 282014	Undetermined (Physical and Ownership)	SD-WAP / 282014
SD-WAP	Jul 24, 1992	SANDIEGOZ / 282014	SD-WAP / 282014	Undetermined (Physical and Ownership)	

Reported By	Date	Type	Identifier	Location	Comments
SD-WAP	Oct 07, 1998	Transponder	0001217E79		
SANDIEGOZ	Jul 02, 1992	Tattoo	SB108		
SANDIEGOZ	Jul 02, 1992	House Name	CONNIE-LENORE		
CINCINNAT	Jun 21, 1988	House Name	LENORE		
CINCINNAT	Jun 21, 1988	Regional Studbook Number	108		Legacy SLocation: UNKNOWN STUDBOOK Legacy Comment: 108 "International Studbook Number" from ARKS 2 records
CINCINNAT	Jun 21, 1988	Studbook Name	SAND12		
CINCINNAT	Jun 21, 1988	Local ID	M5062		
SD-WAP		Local ID	282014		
SANDIEGOZ		Local ID	282014		

Sex Information:

Reported By	Date	Sex	Comments
CINCINNAT	Jun 21, 1988	Female	
SANDIEGOZ	Feb 03, 1982	Female	

Parent Info:

In ZIMS	Parent Info	Type / Probability	Birth Date	Reported By
True	27751557	Dam/100%	Oct 28, 1972	SANDIEGOZ
True	MIG12-26751591	Sire/100%	Dec 12, 1972	SANDIEGOZ

Specimen Report

FATHER OF DAM

ISIS GAN: MIG12-26751591 180343

Pan paniscus Bonobo Pan paniscus

Order: Primates **Family:** Hominidae

Start Date: Jan 01, 1800 **End Date:** Sep 14, 2012

Basic Animal Information

Sex - Contraception: Male -
Birthdate - Age: Dec 12, 1972 - 40Y,9M,3D +/-1Y
Origin: ZAIRE
Birth Type: Wild Born
Sire:
Current Collection: Main Institution Animal Collection
Clutch / Litter:

Status: Pending Confirmation (more than 1 year)
Preferred ID: 180343
Rearing: Parent
Hybrid Status: Not a hybrid
Dam:
Collection Trip:

Physical History:

Reported By	Date	Source / ID	Holder / ID	Term Type	Recipient / ID
STUTTGART	Dec 13, 1973	230903000 /	STUTTGART / 2542	Collected from the Wild(Physical and Ownership)	
SANDIEGOZ	Dec 15, 1980		SANDIEGOZ / 180343	Loan In From (Physical Only)	
STUTTGART	Dec 15, 1980		STUTTGART / 2542	Loan Out To (Initial Transaction - Physical Only)	SANDIEGOZ / 180343
SANDIEGOZ	Jul 10, 1989		SANDIEGOZ / 180343	Loan Transfer To (Physical Only)	SD-WAP / 180343
SD-WAP	Jul 10, 1989		SD-WAP / 180343	Loan In From (Physical Only)	
SD-WAP	Jan 23, 1992		SD-WAP / 180343	Loan Transfer To (Physical Only)	CINCINNAT / 192008
CINCINNAT	Jan 25, 1992		CINCINNAT / 192008	Loan In From (Physical Only)	
SD-WAP	Oct 09, 1994	STUTTGART / 2542	SD-WAP / 180343	Trade From(Physical and Ownership)	
SD-WAP	Oct 09, 1994		SD-WAP / 180343	Loan Out To (Initial Transaction - Physical Only)	CINCINNAT / 192008

Ownership History:

Reported By	Date	Source / ID	Owner / ID	Term Type	Recipient / ID
STUTTGART	Dec 13, 1973	230903000 /	STUTTGART / 2542	Collected from the Wild(Physical and Ownership)	
SANDIEGOZ	Dec 15, 1980		STUTTGART / 2542	Reported Owner	
SD-WAP	Jul 10, 1989		STUTTGART / 2542	Reported Owner	
CINCINNAT	Jan 25, 1992		STUTTGART / 2542	Reported Owner	
SD-WAP	Oct 09, 1994	STUTTGART / 2542	SD-WAP / 180343	Trade From(Physical and Ownership)	
SD-WAP	Oct 09, 1994		SD-WAP / 180343	Reported Owner	
STUTTGART	Oct 10, 1994		STUTTGART / 2542	Trade To (Ownership Only)	SANDIEGOZ / 180343

Reported By	Date	Type	Identifier	Location	Comments
CINCINNAT	Jul 23, 2002	Tattoo	58		Legacy SLocation: rt inner thigh<br>Legacy Comment:
STUTTGART	Nov 05, 1997	Intl Stdbk#	58		
CINCINNAT	Jan 25, 1992	Local ID	192008		
SANDIEGOZ	Dec 15, 1980	Tattoo	SB58		
STUTTGART	Dec 13, 1973	Local ID	2542		
STUTTGART	Dec 13, 1973	House Name	VERNON		
SANDIEGOZ	Dec 13, 1973	House Name	VERNON		
CINCINNAT	Jan 30, 1972	Regional Studbook Number	58		Legacy SLocation: AZA<br>Legacy Comment:
CINCINNAT	Jan 30, 1972	House Name	VERNON		
SANDIEGOZ		Local ID	180343		
SD-WAP		Local ID	180343		

Sex Information:

Reported By	Date	Sex	Comments
CINCINNAT	Jan 25, 1992	Male	
STUTTGART	Dec 13, 1973	Male	

Specimen Report

MOTHER OF DAM

ISIS GAN: 27751557 172284

Pan paniscus Bonobo Pan paniscus

Order: Primates **Family:** Hominidae

Start Date: Jan 01, 1800 **End Date:** Sep 14, 2012

Basic Animal Information

**Sex - Contraception:**	Female - Medical Method - Hormonal/Active	**Status:**	Alive
**Birthdate - Age:**	Oct 28, 1972 - 40Y,10M,17D	**Preferred ID:**	172284
**Origin:**	San Diego Zoo	**Rearing:**	Parent
**Birth Type:**	Captive Born	**Hybrid Status:**	Not a hybrid
**Sire:**		**Dam:**	
**Current Collection:**	Main Institution Animal Collection	**Collection Trip:**	
**Clutch / Litter:**			

Physical History:

Reported By	Date	Source / ID	Holder / ID	Term Type	Recipient / ID
SANDIEGOZ	Oct 28, 1972		SANDIEGOZ / 172284	Birth/Hatch (Physical and Ownership)	
SANDIEGOZ	Jul 11, 1989		SANDIEGOZ / 172284	Undetermined (Physical and Ownership)	SD-WAP / 172284
SD-WAP	Jul 11, 1989	SANDIEGOZ / 172284	SD-WAP / 172284	Undetermined (Physical and Ownership)	
CINCINNAT	Oct 28, 1992		CINCINNAT / 192199	Loan In From (Physical Only)	
SD-WAP	Oct 28, 1992		SD-WAP / 172284	Loan Out To (Initial Transaction - Physical Only)	CINCINNAT / 192199

Ownership History:

Reported By	Date	Source / ID	Owner / ID	Term Type	Recipient / ID
SANDIEGOZ	Oct 28, 1972		SANDIEGOZ / 172284	Birth/Hatch (Physical and Ownership)	
SANDIEGOZ	Jul 11, 1989		SANDIEGOZ / 172284	Undetermined (Physical and Ownership)	SD-WAP / 172284
SD-WAP	Jul 11, 1989	SANDIEGOZ / 172284	SD-WAP / 172284	Undetermined (Physical and Ownership)	
CINCINNAT	Oct 28, 1992		SD-WAP / 172284	Reported Owner	
SD-WAP	Oct 28, 1992		SD-WAP / 172284	Reported Owner	

Reported By	Date	Type	Identifier	Location	Comments
CINCINNAT	Jul 23, 2002	Tattoo	51		Legacy SLocation: lft inner thigh Legacy Comment:
CINCINNAT	Oct 28, 1992	Local ID	192199		
SANDIEGOZ	Oct 28, 1972	House Name	LOUISE		
CINCINNAT	Oct 28, 1972	House Name	LOUISE		
CINCINNAT	Oct 28, 1972	Regional Studbook Number	51		Legacy SLocation: AZA Legacy Comment:
SANDIEGOZ	Oct 28, 1972	Tattoo	SB51		
SANDIEGOZ		Local ID	172284		
SD-WAP		Local ID	172284		

Sex Information:

Reported By	Date	Sex	Comments
CINCINNAT	Oct 28, 1992	Female	
SANDIEGOZ	Oct 28, 1972	Female	

Parent Info:

In ZIMS	Parent Info	Type / Probability	Birth Date	Reported By
True	MIG12-27187805	Dam/100%	Jan 09, 1956	CINCINNAT
True	9041714	Sire/100%	Jan 01, 1958	CINCINNAT

AG06105

AG06105
AG-- 10/12/1982

CC SQC

Print Pedigree Report

Specimen Report

November 18, 2002 8:53:27 AM

International Species Information System

Specimen PHILADELP/100007

Names:

Taxonomic:	Common:	Family:	Order:
Pongo pygmaeus abelii	Sumatran orangutan	Hominidae	Primates

Birth Information:

Sex:	Birth Location:	Birth date:	Birth type:	Hybrid:	Rearing:
Female		~ 1956	Unknown	Not a hybrid	Unknown

Visits:

Date:	Aquisition:	Vendor/LocalID:	Holder:	Disposition:	Recipient/LocalID:	Date:
7 Aug 1962	Purchase from	ZEEHANDLR/UNK	PHILADELP/100007	Sale to	PITTSBURG/000294	10 Mar 1983
11 Mar 1983	Purchase from	PHILADELP/100007	PITTSBURG /294	Death		18 Dec 1986

Identification:

Date:	Type:	Identifier:	Location: Comments:
7 Aug 1962	Global Studbook #	474	474 "International Studbook Number" from ARKS 2 records at PHILADELP
11 Mar 1983	House Name	GOOLAH	at PITTSBURG
28 Oct 1998	Global Studbook #	005055	at PITTSBURG

Sex Information:

Date:	Sex:	Comments:
7 Aug 1962	Female	at PHILADELP
11 Mar 1983	Female	at PITTSBURG

Rearing Information:

Date:	Rearing:	Comments:
7 Aug 1962	Unknown	at PHILADELP
11 Mar 1983	Unknown	at PITTSBURG

Death Information:

Circumstances:	Carcass Disposition:	Carcass recipient:	Necropsy:	Reported By:
Euthanasia	Given to an Institution	UNIV PITT	Urinary, Metabolism	at PITTSBURG

Specimen Report

Print Pedigree Report May 21, 2003

International Species Information System

ISIS Home

Specimen KNOXVILLE/1890

Names:

Taxonomic:	Common:	Family:	Order:
Gorilla gorilla gorilla	Western lowland gorilla	Hominidae	Primates

Birth Information:

Sex:	Birth Location:	Birth date:	Birth type:	Hybrid:	Rearing:
Male	OKLAHOMA	7 Dec 1982	Captive born	Not a hybrid	Parent

Visits:

Date:	Acquisition:	Vendor/LocalID:	Holder:	Disposition:	Recipient/LocalID:	Date:
7 Dec 1982	Birth		OKLAHOMA/082910	Sale to	DENVER/11700	21 Jun 1989
21 Jun 1989	Purchase from	OKLAHOMA/82910	DENVER/11700	Loan Out to	COLO SPRG/94M051	11 Oct 1994
11 Oct 1994	Loan In from	DENVER/11700	COLO SPRG/94M051	Loan Transfer	KNOXVILLE/1890	25 Oct 1999
			DENVER/11700	Loan Out to	KNOXVILLE/1890	27 Oct 1999
27 Oct 1999	Loan In from	DENVER/11700	KNOXVILLE/1890			
16 Apr 2003	Donation from	DENVER/11700	KNOXVILLE/1890			

Identification:

Date:	Type:	Identifier:	Location:	Comments:
7 Dec 1982	House Name	ERNIE		at DENVER
7 Dec 1982	Global Studbook #	808/265		at DENVER
7 Dec 1982	Studbook Name	OKLA 6/G		at DENVER
7 Dec 1982	House Name	ERNIE		at OKLAHOMA
7 Dec 1982	Studbook Name	OKLA 6/G		at OKLAHOMA
7 Dec 1982	Global Studbook #	808		at OKLAHOMA
21 Jun 1989	House Name	ERNIE		at COLO SPRG
21 Jun 1989	Regional Studbook #	808	UNKNOWN STUDBOOK	808 "International Studbook Number" from ARKS 2 records at COLO SPRG
27 Oct 1999	House Name	Ernie		at KNOXVILLE
27 Oct 1999	Regional Studbook #	808	AZA	at KNOXVILLE

Measurements:

Date:	Measurement:	Value:	Units:	Comments:
10 Mar 1986	live animal weight	27.20	kilogram	WKG 27.20 at DENVER
10 Mar 1986	live animal weight	27.20	kilogram	at OKLAHOMA
13 Oct 1987	live animal weight	46.70	kilogram	WKG 46.70 at DENVER
13 Oct 1987	live animal weight	46.70	kilogram	at OKLAHOMA
30 Sep 1991	live animal weight	264.50	pound	WLB 264.50 at DENVER
7 Oct 1991	live animal weight	268.50	pound	WLB 268.50 at DENVER
21 Oct 1991	live animal weight	270.00	pound	WLB 270.00 at DENVER
18 Nov 1991	live animal weight	269.50	pound	WLB 269.50 at DENVER
25 Nov 1991	live animal weight	273.50	pound	WLB 273.50 at DENVER
30 Dec 1991	live animal weight	275.50	pound	WLB 275.50 at DENVER
16 Mar 1992	live animal weight	296.50	pound	WLB 296.50 at DENVER
22 Jun 1992	live animal weight	313.50	pound	WLB 313.50 at DENVER
6 Jul 1992	live animal weight	324.50	pound	WLB 324.50 at DENVER
3 Aug 1992	live animal weight	340.00	pound	WLB 340.00 at DENVER
28 Sep 1992	live animal weight	358.00	pound	WLB 358.00 at DENVER
5 Oct 1992	live animal weight	355.00	pound	WLB 355.00 at DENVER
9 Nov 1992	live animal weight	363.50	pound	WLB 363.50 at DENVER
15 Mar 1993	live animal weight	390.00	pound	WLB 390.00 at DENVER
22 Mar 1993	live animal weight	394.00	pound	WLB 394.00 at DENVER
29 Mar 1993	live animal weight	393.50	pound	WLB 393.50 at DENVER
5 Apr 1993	live animal weight	393.00	pound	WLB 393.00 at DENVER
12 Apr 1993	live animal weight	398.50	pound	WLB 398.50 at DENVER
19 Apr 1993	live animal weight	399.00	pound	WLB 399.00 at DENVER
3 May 1993	live animal weight	405.50	pound	WLB 405.50 at DENVER
10 May 1993	live animal weight	402.00	pound	WLB 402.00 at DENVER
17 May 1993	live animal weight	406.00	pound	WLB 406.00 at DENVER
24 May 1993	live animal weight	411.50	pound	WLB 411.50 at DENVER
14 Jun 1993	live animal weight	409.50	pound	WLB 409.50 at DENVER
28 Jun 1993	live animal weight	416.50	pound	WLB 416.50 at DENVER
5 Jul 1993	live animal weight	421.50	pound	WLB 421.50 at DENVER
12 Jul 1993	live animal weight	421.50	pound	WLB 421.50 at DENVER
19 Jul 1993	live animal weight	425.00	pound	WLB 425.00 at DENVER
26 Jul 1993	live animal weight	424.00	pound	WLB 424.00 at DENVER
18 Oct 1993	live animal weight	441.00	pound	WLB 441.00 at DENVER

25 Oct 1993	live animal weight	440.00	pound	WLB 440.00 at DENVER
15 Nov 1993	live animal weight	433.50	pound	WLB 433.50 at DENVER
22 Nov 1993	live animal weight	434.00	pound	WLB 434.00 at DENVER
29 Nov 1993	live animal weight	432.00	pound	WLB 432.00 at DENVER
6 Dec 1993	live animal weight	435.00	pound	WLB 435.00 at DENVER
13 Dec 1993	live animal weight	437.50	pound	WLB 437.50 at DENVER
27 Dec 1993	live animal weight	443.00	pound	WLB 443.00 at DENVER
3 Jan 1994	live animal weight	445.00	pound	WLB 445.00 at DENVER
17 Jan 1994	live animal weight	442.00	pound	WLB 442.00 at DENVER
31 Jan 1994	live animal weight	442.00	pound	WLB 442.00 at DENVER
14 Feb 1994	live animal weight	441.00	pound	WLB 441.00 at DENVER
20 Feb 1994	live animal weight	438.50	pound	WLB 438.50 at DENVER
28 Feb 1994	live animal weight	438.00	pound	WLB 438.00 at DENVER
7 Mar 1994	live animal weight	442.00	pound	WLB 442.00 at DENVER
14 Mar 1994	live animal weight	444.00	pound	WLB 444.00 at DENVER
21 Mar 1994	live animal weight	446.00	pound	WLB 446.00 at DENVER
29 Mar 1994	live animal weight	440.00	pound	WLB 440.00 at DENVER
4 Apr 1994	live animal weight	438.50	pound	WLB 438.50 at DENVER
11 Apr 1994	live animal weight	436.50	pound	WLB 436.50 at DENVER
18 Apr 1994	live animal weight	437.50	pound	WLB 437.50 at DENVER
9 May 1994	live animal weight	436.00	pound	WLB 436.00 at DENVER
16 May 1994	live animal weight	436.00	pound	WLB 436.00 at DENVER
23 May 1994	live animal weight	429.50	pound	WLB 429.50 at DENVER
30 May 1994	live animal weight	436.00	pound	WLB 436.00 at DENVER
6 Jun 1994	live animal weight	436.00	pound	WLB 436.00 at DENVER
13 Jun 1994	live animal weight	436.00	pound	WLB 436.00 at DENVER
21 Jun 1994	live animal weight	434.00	pound	WLB 434.00 at DENVER
27 Jun 1994	live animal weight	438.00	pound	WLB 438.00 at DENVER
4 Jul 1994	live animal weight	432.00	pound	WLB 432.00 at DENVER
25 Jul 1994	live animal weight	439.00	pound	WLB 439.00 at DENVER
1 Aug 1994	live animal weight	438.00	pound	WLB 438.00 at DENVER
8 Aug 1994	live animal weight	435.50	pound	WLB 435.50 at DENVER
29 Aug 1994	live animal weight	438.00	pound	WLB 438.00 at DENVER
12 Sep 1994	live animal weight	441.00	pound	WLB 441.00 at DENVER
19 Sep 1994	live animal weight	435.50	pound	WLB 435.50 at DENVER
3 Oct 1994	live animal weight	431.00	pound	WLB 431.00 at DENVER
11 Oct 1994	live animal weight	420.00	pound	WLB 420.00 at DENVER
25 Oct 1999	live animal weight	206.40	kilogram	WKG 206.40 at DENVER
25 Oct 1999	live animal weight	206.40	kilogram	Weight from Cheyenne Mt. Zoo at KNOXVILLE
3 Feb 2000	live animal weight	448.00	pound	WLB 448.00 at DENVER
3 Feb 2000	live animal weight	448.00	pound	at KNOXVILLE

Special Information:

Date:	Note:	Comments:
7 Dec 1982	Accession from Group	at DENVER

Sex Information:

Date:	Sex:	Comments:
7 Dec 1982	Male	at DENVER
7 Dec 1982	Male	at OKLAHOMA
11 Oct 1994	Male	at COLO SPRG
27 Oct 1999	Male	at KNOXVILLE

Rearing Information:

Date:	Rearing:	Comments:
7 Dec 1982	Parent	at OKLAHOMA
11 Oct 1994	Unknown	at COLO SPRG
27 Oct 1999	Parent	at KNOXVILLE

Parents:

Date:	Parent type:	ID:	Location:	Comments:
7 Dec 1982	Sire	009701	OKLAHOMA	while at OKLAHOMA
7 Dec 1982	Dam	184403	OKLAHOMA	while at OKLAHOMA

Report Start Date	Specimen Report for OKLAHOMA / 009701	Report End Date
01-Jan-00		12-Jul-11

Taxonomic name: *Gorilla gorilla gorilla* Family: Hominidae

Common name: Western gorilla Order: Primates

Current information

Sex:	Male	Sire ID:	WILD at CAMEROON
Birth type:	Wild Born	Dam ID:	WILD at CAMEROON
Birth Location:	CAMEROON	Rearing:	Unknown
Birthdate-Age:	~ Aug 1960 - ~36Y,8M at death	Hybrid:	Not a hybrid
Time since last Acq:	34Y,5M,6D as of death		

Date in	Acquisition - Vendor/local Id	Holder	Disposition - Recipient/local Id	Date out
28 Nov 1962	Purchase from ZEEHANDLR / NONE	OKLAHOMA / 009701	Death	5 May 1997

Date	Identifier type	Identifier	Location
28 Nov 1962	House Name	Moemba	
28 Nov 1962	Studbook Name	OKLA 11G	
28 Nov 1962	Global Studbook #	144	
28 Sep 2010	Local ID Elsewhere	23903	STOVALL

Date	Type	Value	Units
16 Nov 1979	live animal weight	113.20	kilogram
21 Jun 1982	live animal weight	147.30	kilogram
15 Feb 1984	live animal weight	163.30	kilogram
19 Mar 1985	live animal weight	165.50	kilogram
21 Nov 1985	live animal weight	160.90	kilogram
5 Feb 1986	live animal weight	147.30	kilogram
20 Oct 1987	live animal weight	162.30	kilogram
19 Dec 1988	live animal weight	164.00	kilogram
5 Oct 1990	live animal weight	164.80	kilogram
17 Dec 1991	live animal weight	167.00	kilogram
15 Jun 1993	live animal weight	155.00	kilogram
19 Jan 1995	live animal weight	138.70	kilogram
11 Jun 1996	live animal weight	162.50	kilogram
19 Mar 1997	live animal weight	164.00	kilogram
8 Apr 1997	live animal weight	158.80	kilogram
5 May 1997	live animal weight	148.30	kilogram

SIRE OF PR00107
KNOXVILLE / 1890

Date	Note type	
5 May 1997	Necropsy results	Euthanized - cardiomyopathy<br>Body to Stovall Museum

Date	Enclosure
28 Nov 1962	PR
9 May 1979	GOR
15 Jun 1993	GE
5 May 1997	REMOVED

Date	Sex
28 Nov 1962	Male

Date	Rearing
28 Nov 1962	Unknown

28 Aug 1960	Sire	WILD	CAMEROON
28 Aug 1960	Dam	WILD	CAMEROON

Death Information

According to OKLAHOMA

Circumstances:	Euthanasia
Carcass Disposition	Given to an Institution
Carcass recipient:	SAMNOBLE
Necropsy (Topological):	Cardiovascular
Necropsy (Etiological):	Circulatory, Secondary

Report Start Date
01-Jan-00

Specimen Report for OKLAHOMA / 184403

Report End Date
12-Jul-11

Taxonomic name: *Gorilla gorilla gorilla* — Family: Hominidae

Common name: Western gorilla — Order: Primates

Current information

Sex:	Female	Sire ID:	WILD at CENTRAL AFRICA
Birth type:	Wild Born	Dam ID:	WILD at CENTRAL AFRICA
Birth Location:	CENTRAL AFRICA	Rearing:	Parent
Birthdate-Age:	~ Dec 1962 - 48Y,6M,17D +/-1 Mo.	Hybrid:	Not a hybrid
Time since last Acq:	39Y,7M,8D as of report end date		

Date in	Acquisition - Vendor/local Id	Holder	Disposition - Recipient/local Id	Date out
3 Dec 1971	Purchase from PHILADELP / 10004	OKLAHOMA / 184403		

Date	Identifier type	Identifier	Location
3 Dec 1971	House Name	Kathryn	
3 Dec 1971	Studbook Name	PHIL 31G	
3 Dec 1971	Global Studbook #	140	

DAM OF PR00107
KNOXVILLE/1890

Date	Type	Value	Units	
24 Jun 1964	live animal weight	9.1	kilogram	
28 Mar 1972	live animal weight	77	kilogram	
22 Feb 1984	live animal weight	98	kilogram	
10 Mar 1986	live animal weight	86	kilogram	
13 Oct 1987	live animal weight	92	kilogram	
2 Oct 1990	live animal weight	106	kilogram	
13 Dec 1991	live animal weight	108	kilogram	
18 Aug 1992	live animal weight	96	kilogram	
2 Mar 1993	live animal weight	102	kilogram	
19 May 1993	live animal weight	83	kilogram	
15 Jun 1993	live animal weight	86	kilogram	
1 Jul 1993	live animal weight	84	kilogram	
11 Jun 1996	live animal weight	103	kilogram	
30 May 1997	live animal weight	99	kilogram	
9 May 1998	live animal weight	90	kilogram	
1 Nov 2002	live animal weight	94	kilogram	
2 Mar 2005	live animal weight	108	kilogram	237 lb
10 Apr 2005	live animal weight	106	kilogram	233 lb
17 Jun 2005	live animal weight	96	kilogram	212 lb
14 Oct 2005	live animal weight	93	kilogram	204 lb
30 Dec 2005	live animal weight	97	kilogram	214 lb
4 May 2006	live animal weight	98	kilogram	215 lb
28 Jan 2007	live animal weight	102	kilogram	224 lb
21 Mar 2007	live animal weight	99	kilogram	218 lb
13 Jul 2007	live animal weight	101	kilogram	222 lb
17 Sep 2007	live animal weight	98	kilogram	216 lb
11 Jan 2008	live animal weight	92	kilogram	203 lb
29 May 2008	live animal weight	92	kilogram	202 lb
6 Aug 2008	live animal weight	94	kilogram	206 lb
11 Oct 2008	live animal weight	95	kilogram	210 lb
20 Dec 2008	live animal weight	93	kilogram	205 lb

9 Jul 2010	live animal weight	100	kilogram	
18 Oct 2010	live animal weight	102	kilogram	
26 Jan 2011	live animal weight	103	kilogram	227 lb

Date	Note type	
24 Jun 1964	Acquisition note	Philadelphia received from Zeehandelaar and estimated birth as December 1962 - House name at Philadelphia was Catherine
21 Sep 1998	Physical condition	Left eye removed

Date	Enclosure
3 Dec 1971	GOR
29 Mar 1972	PR
15 Dec 1978	GOR
2 Mar 1993	PR
19 May 1993	GE

Date	Sex	
3 Dec 1971	Female	
23 Oct 2005	Contraception Started	Began birth control pills

Date	Rearing
3 Dec 1971	Parent

Date	Parent(s)	local Id	Location
24 Dec 1962	Sire	WILD	C.AFRICA
24 Dec 1962	Dam	WILD	C.AFRICA

Report Start Date
1/1/1601

Specimen Report for YERKES / 728

Report End Date
1/19/2010

Taxonomic name: *Pan troglodytes* S004933 S004934

Common name: Chimpanzee

Family: Hominidae

Order: Primates

Current information

Sex:	Female	Sire ID:	UNK at Yerkes NPRC, Emory University
Birth type:	Captive Born	Dam ID:	500 at Yerkes NPRC, Emory University
Birth Location:	Yerkes NPRC, Emory University	Rearing:	Parent
Birthdate-Age:	17 May 1995 - 14Y,8M,4D	Hybrid:	Not a hybrid
Time since last Acq:	14Y,8M,4D as of report end date		

Date in	Acquisition - Vendor/local Id	Holder	Disposition - Recipient/local Id	Date out
17 May 1995	Birth	YERKES / 728		

Date	Identifier type	Identifier	Location	Comments
17 May 1995	Global Studbook #	6286		
18 May 1995	House Name	JAIMIE		
18 May 1995	Sire Taxon	Sire amos (495) or phineas (61a)		

Date	Note type	Comments
17 May 1995	National Chimp Plan	ON NCP Breeding Program<br>N/A<br>[[MSS]]
29 Sep 1995	National Chimp Plan	OFF NCP Breeding Program<br>N/A<br>[[MSS]]

Date	Sex	Comments
17 May 1995	Female	

Date	Rearing	Comments
17 May 1995	Parent	

Date	Parent(s)	local Id	Location	Comments
17 May 1995	Sire	UNK	YERKES	
17 May 1995	Dam	500	YERKES	

CORIELL SAMPLE

Report Start Date
1/1/1601

Specimen Report for YERKES / 495

Report End Date
1/19/2010

Taxonomic name: *Pan troglodytes*
Common name: Chimpanzee

S004960

Family: Hominidae
Order: Primates

Current information

Sex:	Male	Sire ID:	61A at Yerkes NPRC, Emory University
Birth type:	Captive Born	Dam ID:	406 at Yerkes NPRC, Emory University
Birth Location:	Yerkes NPRC, Emory University	Rearing:	Unknown
Birthdate-Age:	11 Nov 1981 - 28Y,2M,8D	Hybrid:	Not a hybrid
Time since last Acq:	28Y,2M,8D as of report end date		

Date in	Acquisition - Vendor/local Id	Holder	Disposition - Recipient/local Id	Date out
11 Nov 1981	Birth	YERKES / 495		

Date	Identifier type	Identifier	Location	Comments
17 Nov 1981	House Name	AMOS		
17 Nov 1981	Global Studbook #	4452		

Date	Note type	Comments
30 Sep 1986	National Chimp Plan	ON NCP Breeding Program<br>N/A<br>[[MSS]]

Date	Sex	Comments
11 Nov 1981	Male	

Date	Rearing	Comments
11 Nov 1981	Unknown	

Date	Parent(s)	local Id	Location	Comments
11 Nov 1981	Sire	61A	YERKES	
11 Nov 1981	Dam	406	YERKES	

POSSIBLE FATHER

Specimen Report for YERKES / 61A

Taxonomic name: *Pan troglodytes* S004978 Family: Hominidae
Common name: Chimpanzee S004979 Order: Primates

Current information

Sex:	Male	Sire ID:	
Birth type:	Unknown	Dam ID:	
Birth Location:	Unknown Location	Rearing:	Unknown
Birthdate-Age:	~ 1966 - ~44Y	Hybrid:	Not a hybrid
Time since last Acq:	36Y,3M,27D as of report end date		

Date in	Acquisition - Vendor/local Id	Holder	Disposition - Recipient/local Id	Date out
22 Sep 1973	Donation from HAWTHORNE / UNK	YERKES / 61A		

Date	Identifier type	Identifier	Location	Comments
22 Sep 1973	House Name	PHINEAS		
22 Sep 1973	Global Studbook #	1606		

Date	Note type	Comments
30 Sep 1986	National Chimp Plan	ON NCP Breeding Program<br>N/A<br>[[MSS]]

Date	Sex	Comments
22 Sep 1973	Male	

Date	Rearing	Comments
22 Sep 1973	Unknown	

POSSIBLE PATERNAL GRANDFATHER

Specimen Report for YERKES / 406

Taxonomic name: *Pan troglodytes* — Family: Hominidae

Common name: Chimpanzee — Order: Primates

Current Information

Sex:	Female	Sire ID:	
Birth type:	Unknown	Dam ID:	
Birth Location:		Rearing:	Unknown
Birthdate-Age:	~ 1959 - ~41Y at death	Hybrid:	Not a hybrid
Time since last Acq:	31Y,5M,22D as of death		

Date in	Acquisition - Vendor/local Id	Holder	Disposition - Recipient/local Id	Date out
13 Nov 1969	Trade from HOLLOMAN / 122	YERKES / 406	Death	6 May 2001

Date	Identifier type	Identifier	Location	Comments
13 Nov 1969	House Name	SONIA		
13 Nov 1969	Global Studbook #	1040		

Date	Note type	Comments
12 Feb 1990	National Chimp Plan	ON NCP Breeding Program
29 Sep 1995	National Chimp Plan	OFF NCP Breeding Program<br>N/A<br>[[MSS]]

Date	Sex	Comments
13 Nov 1969	Female	

Date	Rearing	Comments
13 Nov 1969	Unknown	

POSSIBLE PATERNAL GRANDMOTHER

Specimen Report for YERKES / 61A

Taxonomic name: *Pan troglodytes*
Common name: Chimpanzee

S004978
S004979

Family: Hominidae
Order: Primates

Current information

Sex:	Male	Sire ID:	
Birth type:	Unknown	Dam ID:	
Birth Location:	Unknown Location	Rearing:	Unknown
Birthdate-Age:	~ 1966 - ~44Y	Hybrid:	Not a hybrid
Time since last Acq:	36Y,3M,27D as of report end date		

Date in	Acquisition - Vendor/local Id	Holder	Disposition - Recipient/local Id	Date out
22 Sep 1973	Donation from HAWTHORNE / UNK	YERKES / 61A		

Date	Identifier type	Identifier	Location	Comments
22 Sep 1973	House Name	PHINEAS		
22 Sep 1973	Global Studbook #	1606		

Date	Note type	Comments
30 Sep 1986	National Chimp Plan	ON NCP Breeding Program<br>N/A<br>[[MSS]]

Date	Sex	Comments
22 Sep 1973	Male	

Date	Rearing	Comments
22 Sep 1973	Unknown	

POSSIBLE FATHER

Report Start Date 1/1/1601	Specimen Report for YERKES / 500	Report End Date 1/19/2010

Taxonomic name: *Pan troglodytes*   S005051   Family: Hominidae

Common name: Chimpanzee   Order: Primates

Current information

Sex:	Female	Sire ID:	
Birth type:	Wild Born	Dam ID:	
Birth Location:		Rearing:	Unknown
Birthdate-Age:	~ Oct 1973 - ~36Y,2M	Hybrid:	Not a hybrid
Time since last Acq:	35Y,5M,0D as of report end date		

Date in	Acquisition - Vendor/local Id	Holder	Disposition - Recipient/local Id	Date out
20 Aug 1974	Donation from LUCAS E / SUE	YERKES / 500		

Date	Identifier type	Identifier	Location	Comments
20 Aug 1974	House Name	ERICKA		
20 Aug 1974	Global Studbook #	3140		

Date	Note type	Comments
30 Sep 1986	National Chimp Plan	ON NCP Breeding Program<br>N/A<br>[[MSS]]

Date	Sex	Comments
20 Aug 1974	Female	

Date	Rearing	Comments
20 Aug 1974	Unknown	

MOTHER

Specimen Report for YERKES / 597

Taxonomic name: *Pan troglodytes* S003648 S003649 Family: Hominidae
Common name: Chimpanzee Order: Primates

Current information

Sex:	Male	Sire ID:	389 at Yerkes NPRC, Emory University
Birth type:	Captive Born	Dam ID:	46A at Yerkes NPRC, Emory University
Birth Location:	Yerkes NPRC, Emory University	Rearing:	Hand
Birthdate-Age:	27 Aug 1990 - 19Y,10M,26D	Hybrid:	Not a hybrid
Time since last Acq:	19Y,10M,26D as of report end date		

Date in	Acquisition - Vendor/local Id	Holder	Disposition - Recipient/local Id	Date out
27 Aug 1990	Birth	YERKES / 597		

Date	Identifier type	Identifier	Location	Comments
27 Aug 1990	House Name	JOSH		
27 Aug 1990	Global Studbook #	5678		

Date	Note type	Comments
27 Aug 1990	National Chimp Plan	ON NCP Breeding Program
30 Mar 1996	National Chimp Plan	OFF NCP Breeding Program<br>N/A<br>[[MSS]]

Date	Sex	Comments
27 Aug 1990	Male	

Date	Rearing	Comments
27 Aug 1990	Hand	

Date	Parent(s)	local Id	Location	Comments
27 Aug 1990	Sire	389	YERKES	
27 Aug 1990	Dam	46A	YERKES	

CORIELL SAMPLE

Specimen Report for YERKES / 389

Taxonomic name: *Pan troglodytes*
Common name: Chimpanzee

S004968

Family: Hominidae
Order: Primates

Current information

Sex:	Male	Sire ID:	
Birth type:	Unknown	Dam ID:	
Birth Location:		Rearing:	Unknown
Birthdate-Age:	~ 1969 - ~41Y	Hybrid:	Not a hybrid
Time since last Acq:	35Y,10M,14D as of report end date		

Date in	Acquisition - Vendor/local Id	Holder	Disposition - Recipient/local Id	Date out
7 Mar 1974	Donation from DURHAM / UNK	YERKES / 389		

Date	Identifier type	Identifier	Location	Comments
7 Mar 1974	House Name	ROGGER		
7 Mar 1974	Global Studbook #	2363		

Date	Note type	Comments
30 Sep 1995	National Chimp Plan	ON NCP Breeding Program<br>N/A<br>[[MSS]]

Date	Sex	Comments
7 Mar 1974	Male	

Date	Rearing	Comments
7 Mar 1974	Unknown	

FATHER

Report Start Date
1/1/1601

Specimen Report for YERKES / 46A

Report End Date
1/19/2010

S003639

Taxonomic name: *Pan troglodytes*
Common name: Chimpanzee

Family: Hominidae
Order: Primates

Current information

Sex:	Female	Sire ID:	
Birth type:	Unknown	Dam ID:	
Birth Location:		Rearing:	Unknown
Birthdate-Age:	~ 1967 - ~43Y	Hybrid:	Not a hybrid
Time since last Acq:	36Y,3M,27D as of report end date		

Date in	Acquisition - Vendor/local Id	Holder	Disposition - Recipient/local Id	Date out
22 Sep 1973	Purchase from LEMSIP / 46	YERKES / 46A		

Date	Identifier type	Identifier	Location	Comments
22 Sep 1973	House Name	GAY		
22 Sep 1973	Global Studbook #	2208		

Date	Note type	Comments
12 Feb 1990	National Chimp Plan	ON NCP Breeding Program

Date	Sex	Comments
22 Sep 1973	Female	

Date	Rearing	Comments
22 Sep 1973	Unknown	

MOTHER

Report Start Date
1/1/1601

Specimen Report for YERKES / 643

Report End Date
1/19/2010

Taxonomic name: *Pan troglodytes* S003611
Common name: Chimpanzee

Family: Hominidae
Order: Primates

Current information

Sex:	Male	Sire ID:	381 at Yerkes NPRC, Emory University
Birth type:	Captive Born	Dam ID:	434 at Yerkes NPRC, Emory University
Birth Location:	Yerkes NPRC, Emory University	Rearing:	Parent
Birthdate-Age:	26 Sep 1993 - 16Y,3M,23D	Hybrid:	Not a hybrid
Time since last Acq:	16Y,3M,23D as of report end date		

Date in	Acquisition - Vendor/local Id	Holder	Disposition - Recipient/local Id	Date out
26 Sep 1993	Birth	YERKES / 643		

Date	Identifier type	Identifier	Location	Comments
26 Sep 1993	House Name	MAVERICK		
26 Sep 1993	Global Studbook #	6272		

Date	Note type	Comments
26 Sep 1993	National Chimp Plan	ON NCP Breeding Program
29 Sep 1995	National Chimp Plan	OFF NCP Breeding Program<br>N/A<br>[[MSS]]

Date	Sex	Comments
26 Sep 1993	Male	

Date	Rearing	Comments
26 Sep 1993	Parent	

Date	Parent(s)	local Id	Location	Comments
26 Sep 1993	Sire	381	YERKES	
26 Sep 1993	Dam	434	YERKES	

CORIELL SAMPLE

Report Start Date		Report End Date
1/1/1601	# Specimen Report for YERKES / 381	1/19/2010

Taxonomic name: *Pan troglodytes* S003657 Family: Hominidae
Common name: Chimpanzee Order: Primates

Current information

Sex:	Male	Sire ID:	
Birth type:	Unknown	Dam ID:	
Birth Location:		Rearing:	Unknown
Birthdate-Age:	~ 1964 - ~46Y	Hybrid:	Not a hybrid
Time since last Acq:	37Y,3M,23D as of report end date		

Date in	Acquisition - Vendor/local Id	Holder	Disposition - Recipient/local Id	Date out
26 Sep 1972	Donation from DURHAM / UNK	YERKES / 381		

Date	Identifier type	Identifier	Location	Comments
26 Sep 1972	House Name	JIMOH		
26 Sep 1972	Global Studbook #	1629		

Date	Note type	Comments
30 Sep 1986	National Chimp Plan	ON NCP Breeding Program<br>N/A<br>[[MSS]]
29 Sep 1995	National Chimp Plan	OFF NCP Breeding Program<br>N/A<br>[[MSS]]

Date	Sex	Comments
26 Sep 1972	Male	

Date	Rearing	Comments
26 Sep 1972	Unknown	

FATHER

Report Start Date
1/1/1601

Specimen Report for YERKES / 434

Report End Date
1/19/2010

Taxonomic name: *Pan troglodytes* S003612
Common name: Chimpanzee

Family: Hominidae
Order: Primates

Current Information

Sex:	Female	Sire ID:	UNK at Yerkes NPRC, Emory University
Birth type:	Captive Born	Dam ID:	268 at Yerkes NPRC, Emory University
Birth Location:	Yerkes NPRC, Emory University	Rearing:	Unknown
Birthdate-Age:	13 Jan 1971 - 29Y,9M,9D at death	Hybrid:	Not a hybrid
Time since last Acq:	29Y,9M,9D as of death		

Date in	Acquisition - Vendor/local Id	Holder	Disposition - Recipient/local Id	Date out
13 Jan 1971	Birth	YERKES / 434	Death / NHXX	23 Oct 2000

Date	Identifier type	Identifier	Location	Comments
13 Jan 1971	House Name	MARILYNE		
13 Jan 1971	Global Studbook #	2831		

Date	Note type	Comments
30 Sep 1986	National Chimp Plan	ON NCP Breeding Program<br>N/A<br>[[MSS]]

Date	Sex	Comments
13 Jan 1971	Female	

Date	Rearing	Comments
13 Jan 1971	Unknown	

Date	Parent(s)	local Id	Location	Comments
13 Jan 1971	Sire	UNK	YERKES	
13 Jan 1971	Dam	268	YERKES	

Death Information

According to YERKES

Circumstances:	Other/Unknown
Carcass Disposition	Unknown
Carcass recipient:	
Necropsy (Topological):	Unknown
Necropsy (Etiological):	Unknown

MOTHER

PR01109
PR-- 11/05/2004
CC SOC

Specimen BROWNSVIL/71

Names:

Taxonomic:	Common:	Family:	Order:
Pongo pygmaeus	**Sumatran orangutan**	Hominidae	Primates

Birth Information:

Sex:	Birth Location:	Birth date:	Birth type:	Hybrid:	Rearing:
Female	JAJASAN	~ 1964	Wild Born	Not a hybrid	Unknown

Visits:

Date:	Acquisition:	Vendor/LocalID:	Reported by:	Disposition:	Recipient/LocalID:	Date:
13 Oct 1969	Loan In from	BROWNSVIL/71	OMAHA/738	Loan Return to Owner	BROWNSVIL/71	17 Jul 1970
19 Jul 1970	Purchase from	THOU OAKS/UNK	BROWNSVIL/71			

Identification:

Date:	Type:	Identifier:	Location:	Comments:
12 Oct 1964	Regional Studbook #	1044	UNKNOWN STUDBOOK	1044 UNKNOWN STUDBOOK "International Studbook Number" from ARKS 2 records at OMAHA
13 Oct 1969	House Name	SUSIE		at OMAHA
19 Jul 1970	Global Studbook #	1044		at BROWNSVIL
19 Jul 1970	House Name	SUSIE		at BROWNSVIL
20 May 1986	Global Studbook #	1044		at BROWNSVIL
20 May 1993	Transponder ID	00-00F7-1D63	Craniomedial to	at BROWNSVIL
30 Aug 1995	Tag/Band	PIT 00004EBBF1 CAUDOMEDIAL TO R SC	CAUDOMEDIAL TO R	at BROWNSVIL
28 Jan 1999	Tattoo	PIT 0001FD9766 IN MGA IMPLANT	IN MGA IMPLANT	at BROWNSVIL
15 Jan 2004	Transponder ID	00-01EE-5B42	In MGA implant	at BROWNSVIL

Sex Information:

Date:	Sex:	Comments:
13 Oct 1969	Female	at OMAHA
19 Jul 1970	Female	at BROWNSVIL

Rearing Information:

Date:	Rearing:	Comments:
13 Oct 1969	Unknown	at OMAHA

Parents:

Date:	Parent type:	ID:	Location:	Comments:
-	Sire	Unknown	Wild	-
-	Dam	Unknown	Wild	-

Specimen Report

Print Pedigree Report March 13, 2003

International Species Information System

ISIS Home

PR00572
PR - - 03/14/2003
BU SQC

Specimen SANDIEGOZ/027944

Names:

Taxonomic:	Common:	Family:	Order:
Gorilla gorilla gorilla	Western lowland gorilla	Hominidae	Primates

Birth Information:

Sex:	Birth Location:	Birth date:	Birth type:	Hybrid:	Rearing:
Male	AFRICAN	~ Jan 1969	Wild Born		Unknown

Visits:

Date:	Acquisition:	Vendor/LocalID:	Holder:	Disposition:	Recipient/LocalID:	Date:
28 Mar 1984	Donation from	WEYBRIDGE	SANDIEGOZ/027944	Loan Out to	PHILADELP/101834	29 Jun 1989
30 Jun 1989	Loan In from	SANDIEGOZ/027944	PHILADELP/101834	Loan Return to Owner	SANDIEGOZ/024680	16 Feb 1991
16 Feb 1991	Loan Returned to Us	PHILADELP/101834	SANDIEGOZ/027944			

PR00573
PR - - 03/14/2003
CC SQC

Identification:

Date:	Type:	Identifier:	Location:	Comments:
5 Jul 1969	House Name	MEMBA		at PHILADELP
5 Jul 1969	Regional Studbook #	442	AZA	at PHILADELP
5 Jul 1969	Studbook Name	WEY 4/G		at PHILADELP
5 Jul 1969	Global Studbook #	442		at PHILADELP
16 Feb 1991	House Name	MEMBA		at SANDIEGOZ

Sex Information:

Date:	Sex:	Comments:
1 Jan 1969	Male	at SANDIEGOZ
30 Jun 1989	Male	at PHILADELP

Rearing Information:

Date:	Rearing:	Comments:
30 Jun 1989	Unknown	at PHILADELP

Parents:

Date:	Parent type:	ID:	Location:	Comments:
-	Sire	Unknown	Wild	-
-	Dam	Unknown	Wild	-

Specimen Report

Print Pedigree Report May 28, 2003

International Species Information System

ISIS Home

Specimen SANDIEGOZ/174013

Names:

Taxonomic:	Common:	Family:	Order:
Pan paniscus	Pygmy chimpanzee	Hominidae	Primates

Birth Information:

Sex:	Birth Location:	Birth date:	Birth type:	Hybrid:	Rearing:
Female	SANDIEGOZ	22 Jan 1974	Captive born		

Visits:

Date:	Acquisition:	Vendor/LocalID:	Holder:	Disposition:	Recipient/LocalID:	Date:
22 Jan 1974	Birth		SANDIEGOZ/174013	Term Free Disposition	SD-WAP/174013	10 Jul 1989
10 Jul 1989	Term Free Acquisition	SANDIEGOZ/174013	SD-WAP/174013			

Identification:

Date:	Type:	Identifier:	Location:	Comments:
22 Jan 1974	House Name	LORETTA		at SANDIEGOZ
22 Jan 1974	Tattoo	SB59		at SANDIEGOZ
26 Oct 1998	Transponder ID	000132ABB7		at SD-WAP

Sex Information:

Date:	Sex:	Comments:
22 Jan 1974	Female	at SD-WAP
22 Jan 1974	Female	at SANDIEGOZ

Parents:

Date:	Parent type:	ID:	Location:	Comments:
22 Jan 1974	Sire	160134	SANDIEGOZ	while at SANDIEGOZ
22 Jan 1974	Dam	162184	SANDIEGOZ	while at SANDIEGOZ

PR00230
PR-- 07/2/2002
CC

Specimen Report

Print Pedigree Report June 6, 2003

International Species Information System

ISIS Home

Specimen SD-WAP/165151

Names:

Taxonomic:	Common:	Family:	Order:
Gorilla gorilla gorilla	Western lowland gorilla	Hominidae	Primates

Birth Information:

Sex:	Birth Location:	Birth date:	Birth type:	Hybrid:	Rearing:
Female	SANDIEGOZ	3 Jun 1965	Captive born		Unknown

Visits:

Date:	Acquisition:	Vendor/LocalID:	Holder:	Disposition:	Recipient/LocalID:	Date:
16 Apr 1972	Term Free Acquisition	SANDIEGOZ/165151	SD-WAP/165151	Loan Out to	FRESNO/1082	1 Mar 1978
1 Mar 1978	Loan In from	SD-WAP/165151	FRESNO/1082	Loan Return to Owner	SD-WAP/100087	12 Jul 1981
			SD-WAP/165151	Term Free Disposition	SANDIEGOZ/165151	11 Jul 1981

Identification:

Date:	Type:	Identifier:	Location:	Comments:
1 Mar 1978	House Name	ALVILA		at FRESNO
1 Mar 1978	Regional Studbook #	223/7	AZA	at FRESNO

Sex Information:

Date:	Sex:	Comments:
3 Jun 1965	Female	at SD-WAP
1 Mar 1978	Female	at FRESNO

Rearing Information:

Date:	Rearing:	Comments:
1 Mar 1978	Unknown	at FRESNO

Specimen Report

Print Pedigree Report May 21, 2003

International Species Information System

ISIS Home

Specimen JACKSONVL/898051

Names:

Taxonomic:	Common:	Family:	Order:
Pan paniscus	Pygmy chimpanzee	Hominidae	Primates

Birth Information:

Sex:	Birth Location:	Birth date:	Birth type:	Hybrid:	Rearing:
Male	KINSHA.CT	1 Jan 1971	Wild Born		Parent

Visits:

Date:	Acquisition:	Vendor/LocalID:	Holder:	Disposition:	Recipient/LocalID:	Date:
27 Mar 1975	Donation from	KINSHASA/UNK	YERKES/P4	Loan Out to	JACKSONVL	15 Apr 1998
15 Apr 1998	Loan In from	YERKES/P4	JACKSONVL/898051			
			YERKES/P4	Donation to	JACKSONVL	27 Jul 1999
7 Sep 1999	Donation from	YERKES/P4	JACKSONVL/898051			

Identification:

Date:	Type:	Identifier:	Location:	Comments:
27 Mar 1975	House Name	BOSONDJO		at YERKES
27 Mar 1975	Global Studbook #	64		at YERKES
27 Mar 1975	House Name	BOSONDJO		at JACKSONVL
27 Mar 1975	Global Studbook #	64		at JACKSONVL

Measurements:

Date:	Measurement:	Value:	Units:	Comments:
18 Jul 1998	live animal weight	58.57	kilogram	at JACKSONVL
25 Jul 1998	live animal weight	57.66	kilogram	at JACKSONVL
1 Aug 1998	live animal weight	57.20	kilogram	at JACKSONVL
29 Aug 1998	live animal weight	60.00	kilogram	at JACKSONVL
2 Oct 1998	live animal weight	54.60	kilogram	at JACKSONVL
19 Dec 1998	live animal weight	52.20	kilogram	at JACKSONVL
21 May 1999	live animal weight	56.70	kilogram	at JACKSONVL
25 Jun 1999	live animal weight	52.36	kilogram	at JACKSONVL
24 Nov 2000	live animal weight	55.57	kilogram	at JACKSONVL
27 Apr 2002	live animal weight	54.90	kilogram	at JACKSONVL

Sex Information:

Date:	Sex:	Comments:
1 Jan 1971	Male	at YERKES
15 Apr 1998	Male	at JACKSONVL

Rearing Information:

Date:	Rearing:	Comments:
15 Apr 1998	Parent	at JACKSONVL

Parents:

Date:	Parent type:	ID:	Location:	Comments:
15 Apr 1998	Sire	WILD	ZAIRE	while at JACKSONVL
15 Apr 1998	Dam	WILD	ZAIRE	while at JACKSONVL

PR00054/146/1003

Print Pedigree Report

ISIS International Species Information System

Specimen SANDIEGOZ/595117

Names:

Taxonomic:	Common:	Family:	Order:
Pongo pygmaeus abelii	**Sumatran orangutan**	Hominidae	Primates

Birth Information:

Sex:	Birth Location:	Birth date:	Birth type:	Hybrid:	Rearing:
Male	SANDIEGOZ	26 Mar 1995	Captive born		Unknown

Visits:

Date:	Aquisition:	Vendor/LocalID:	Holder:	Disposition:	Recipient/LocalID:	Date:
26 Mar 1995	Birth, Loan In	SACRAMNTO/100934	SANDIEGOZ/595117			
26 Mar 1995	Birth, Loan Out	SANDIEGOZ/595117	SACRAMNTO/100934			

Identification:

Date:	Type:	Identifier:	Location:	Comments:
26 Mar 1995	House Name	SATU		at SANDIEGOZ
26 Mar 1995	Transponder ID	000133CD31		at SANDIEGOZ

Sex Information:

Date:	Sex:	Comments:
26 Mar 1995	Male	at SANDIEGOZ
26 Mar 1995	Male	at SACRAMNTO

Rearing Information:

Date:	Rearing:	Comments:
26 Mar 1995	Unknown	at SACRAMNTO

Parents:

Date:	Parent type:	ID:	Location:	Comments:
26 Mar 1995	Sire	594030	SANDIEGOZ	while at SANDIEGOZ
26 Mar 1995	Sire	100759	COLO SPRG	while at SACRAMNTO
26 Mar 1995	Dam	100084	SACRAMNTO	while at SACRAMNTO
26 Mar 1995	Dam	593036	SANDIEGOZ	while at SANDIEGOZ

SIPE

Specimen Report

Print Pedigree Report

International Species Information System

9 May 2012

Specimen SANDIEGOZ/594030; COLO SPRG/100759; FRESNO/1573; SD-WAP/594030; ROLLING H/110512

Names:

Taxonomic:	Common:	Family:	Order:
Pongo pygmaeus abelii	Sumatran orangutan	Hominidae	Primates

Birth Information:

Sex:	Birth Location:	Birth date:	Birth type:	Hybrid:	Rearing:
Male	COLO SPRG	23 Aug 1976	Captive born	Not a hybrid	Unknown

Visits:

Date:	Acquisition:	Vendor/LocalID:	Reported by:	Disposition:	Recipient/LocalID:	Date:
23 Aug 1976	Birth		COLO SPRG/100759	Loan Out to	FRESNO/1573	10 Jun 1987
11 Jun 1987	Loan In from	COLO SPRG/100759	FRESNO/1573	Loan Transfer	SD-WAP/594030	26 Jan 1994
			COLO SPRG/100759	Loan Out to	SANDIEGOZ/594030	26 Jan 1994
26 Jan 1994	Loan In from	COLO SPRG/100759	SANDIEGOZ/594030			
			SANDIEGOZ/594030	Loan Transfer	SD-WAP/594030	4 Sep 2002
4 Sep 2002	Loan In from	COLO SPRG/100759	SD-WAP/594030			
			SD-WAP/594030	Loan Transfer	SANDIEGOZ/594030	2 Apr 2003
2 Apr 2003	Loan In from	COLO SPRG/100759	SANDIEGOZ/594030			
			COLO SPRG/100759	Loan Out to	ROLLING H/110512	18 Apr 2011
18 Apr 2011	Loan In from	COLO SPRG/100759	ROLLING H/110512			
18 Apr 2011	Donation from	COLO SPRG/100759	ROLLING H/110512			
			COLO SPRG/100759	Donation to	ROLLING H/110512	18 Apr 2011

Identification:

Date:	Type:	Identifier:	Location:	Comments:
23 Aug 1976	House Name	CLYDE		at FRESNO
23 Aug 1976	Global Studbook #	1529		1529 "International Studbook Number" from ARKS 2 records at FRESNO
23 Aug 1976	Studbook Name	CLYDE		at FRESNO
23 Aug 1976	House Name	CLYDE		at COLO SPRG
23 Aug 1976	Global Studbook #	1529		1529 "International Studbook Number" from ARKS 2 records at COLO SPRG
23 Aug 1976	Global Studbook #	1529		"International Studbook Number" from ARKS 2 records (from COLO SPRG specimen report) at ROLLING H
11 Jun 1987	Old Accession #	100759		Old Accession number 100759 from Cheyenne Mountain Zoo. at FRESNO
1 Apr 1993	Regional Studbook #	721	ASMP	at FRESNO
26 Jan 1994	House Name	CLYDE		at SANDIEGOZ
12 May 2011	House Name	Clyde		at ROLLING H

Measurements:

Date:	Measurement:	Value:	Units:	Comments:
12 Jun 1987	live animal weight	152	pound	at FRESNO
22 Jun 1992	live animal weight	236.00	pound	236 LB at FRESNO
25 Jan 1994	live animal weight	2821.0	pound	2821 LBS M at FRESNO
15 Jun 2005	live animal weight	130	kilogram	as recorded on Health Problem List from San Diego Zoo. at ROLLING H
8 May 2011	live animal weight	140	kilogram	as recorded on ADT filled out by SDZ. at ROLLING H

Sex Information:

Date:	Sex:	Comments:
23 Aug 1976	Male	at SD-WAP
23 Aug 1976	Male	at SANDIEGOZ
23 Aug 1976	Male	at COLO SPRG
11 Jun 1987	Male	at FRESNO

11 May 2011	Male	at ROLLING H

Rearing Information:

Date:	Rearing:	Comments:
23 Aug 1976	Unknown	at COLO SPRG
11 Jun 1987	Unknown	at FRESNO
11 May 2011	Unknown	at ROLLING H

Parents:

Date:	Parent type:	ID:	Location:	Comments:
23 Aug 1976	Sire	100220	COLO SPRG	while at COLO SPRG
23 Aug 1976	Dam	100222	COLO SPRG	while at COLO SPRG

DAM -
WILD CAUGHT

Specimen Report

Print Pedigree Report

International Species Information System 9 May 2012

Specimen SANDIEGOZ/593036; SACRAMNTO/100084; SD-WAP/593036

Names:

Taxonomic:	Common:	Family:	Order:
Pongo pygmaeus abelii	Sumatran orangutan	Hominidae	Primates

Birth Information:

Sex:	Birth Location:	Birth date:	Birth type:	Hybrid:	Rearing:
Female	SUMATRA	~ 1960	Wild Born	Not a hybrid	Unknown

Visits:

Date:	Acquisition:	Vendor/LocalID:	Reported by:	Disposition:	Recipient/LocalID:	Date:
30 Jun 1965	Purchase from	GOEBEL G/NONE	SACRAMNTO/100084	Loan Out to	SANDIEGOZ/593036	14 Feb 1993
15 Feb 1993	Loan In from	SACRAMNTO/100084	SANDIEGOZ/593036	Loan Transfer	SD-WAP/593036	3 Sep 2002
3 Sep 2002	Loan In from	SACRAMNTO/100084	SD-WAP/593036	Loan Transfer	SANDIEGOZ/593036	2 Apr 2003
2 Apr 2003	Loan In from	SACRAMNTO/100084	SANDIEGOZ/593036			
			SACRAMNTO/100084	Death		17 Jan 2008
			SANDIEGOZ/593036	Death		17 Jan 2008

Identification:

Date:	Type:	Identifier:	Location:	Comments:
29 Jun 1960	Regional Studbook #	733	AZA	at SACRAMNTO
30 Jun 1965	House Name	Josephine		at SACRAMNTO
15 Feb 1993	House Name	JOSEPHINE		at SANDIEGOZ
15 Feb 1993	Tattoo	733		at SANDIEGOZ

Sex Information:

Date:	Sex:	Comments:
1 Jan 1960	Female	at SD-WAP
1 Jan 1960	Female	at SANDIEGOZ
30 Jun 1965	Female	at SACRAMNTO

Rearing Information:

Date:	Rearing:	Comments:
30 Jun 1965	Unknown	at SACRAMNTO

Parents:

Date:	Parent type:	ID:	Location:	Comments:
29 Jun 1960	Sire	WILD	SUMATRA	while at SACRAMNTO
29 Jun 1960	Dam	WILD	SUMATRA	while at SACRAMNTO

Death information:

Circumstances:	Carcass Disposition:	Carcass recipient:	Necropsy:	Reported By:
Other/Unknown	Unknown		Unknown, Unknown	at SACRAMNTO
Euthanasia	Given to an Institution	LA MUS	Cardiovascular, Mechanical Abnormality	at SANDIEGOZ
