# Supplementary material for: Conservation of cell-intrinsic immune responses in diverse nonhuman primate species
Source: Life Sci Alliance. 2019 Oct 24;2(5):e201900495. doi: 10.26508/lsa.201900495 (PMC6814850; doi:10.26508/lsa.201900495)
Supplement: Supplementary file 6 [file LSA-2019-00495_Supplemental_Data_3.zip › DatasetS3/README_DatasetS3.rtf]

These files represent the differential gene expression (poly(I:C)-transfected versus mock-transfected) outputs for each of the species. For all the primate species (see information about the mouse samples below), the reads were mapped to the human genome (denoted in the filenames by “HumanMapped_DGE”). These reads were then filtered on a species-by-species basis to the ENSEMBL IDs that have a listed one-to-one human ortholog. To make comparisons appropriately, for every NHP species there is a human DGE profile limited to the same ENSEMBL IDs as the NHP species in question. Hence there are 8 NHP DGE profiles (denoted in file name as “NHPspecies.treatmenttreated dds1”) + 8 human DGE profiles (denoted in file name as (“human_treated_v_mock_related_NHP species”).In performing the DESeq2 analysis, the design used to model the samples and generate our “dds” object was  ~species + species:donor.n + treatment + species:treatment (see Github markdown files for code in complete context).  The reference treatment level was mock-transfected and the reference species human. Thus, to get the DGE profiles for each species, we set up our contrast so that we added the human (i.e. main effect) to the interaction term of our "speciesX.treatmenttreated." In our code, this corresponded to the third and eighth entries in our resultsNames(dds). For the mouse samples: the reads were aligned to the mouse genome, so all the DGE profiles for mouse were done with the murine annotations. Thus, there is no “related” human file for the mouse DGEs. Since the DESeq2 analysis was being done on just mouse without any “related” human samples, the design for modeling the samples was “simpler”: ~donor + treatment
