# Supplementary material for: Conservation of cell-intrinsic immune responses in diverse nonhuman primate species
Source: Life Sci Alliance. 2019 Oct 24;2(5):e201900495. doi: 10.26508/lsa.201900495 (PMC6814850; doi:10.26508/lsa.201900495)
Supplement: Supplementary file 8 [file LSA-2019-00495_Supplemental_Data_5.zip › DatasetS5/README_DatasetS5.rtf]

These files represent the differential gene expression of each NHP species relative to the human DGE profile in response to poly(I:C) treatment. The reads were mapped to the human or species-specific genome (denoted in the filenames by either “HumanMapped_DGE” or “SpeciesMapped_DGE”, respectively). These reads were then filtered on a species-by-species basis to the ENSEMBL IDs that have a listed one-to-one human ortholog. In performing the DESeq2 analysis, the design used to model the samples and generate our “dds” object was  ~species + species:donor.n + treatment + species:treatment (see Github markdown files for code in complete context).  The reference treatment level was mock-transfected and the reference species human. Thus, to get a DGE profile that told us how each NHP species differed from human in the response to poly(I:C), the contrast used was name = resultsNames_output_dds1[8] which corresponds to “treatment_treated” for the reference species, human. 
