# Supplementary material for: Conservation of cell-intrinsic immune responses in diverse nonhuman primate species
Source: Life Sci Alliance. 2019 Oct 24;2(5):e201900495. doi: 10.26508/lsa.201900495 (PMC6814850; doi:10.26508/lsa.201900495)
Supplement: Supplementary file 9 [file LSA-2019-00495_Supplemental_Data_6.zip › DatasetS6/README_DatasetS6.rtf]

These files give the log2FoldChange values (poly(I:C) transfected vs mock transfected) for the genes either uniquely differentially expressed compared to human upon poly(I:C) treatment in a single NHP species or in a group of NHP species (i.e. Old World Monkeys). Note that the genes identified as being differentially expressed compared to human were determined as a result of the UpSet analysis shown in Figure 3, where candidate genes were those that had an absolute(log2FoldChange) >= 3 at a significance value of padj <= 0.05. The expression of these candidate genes was then determined for human samples (poly(I:C) transfected vs mock) and then for each NHP species (poly(I:C) transfected vs mock) in which these candidate genes had first been “flagged.” These expression data are shown in the heat maps in Figure 4 and Figure S10. As with the other large datasets, the genome the reads were originally mapped to is indicated in the file name by “HumanMapped” or “SpeciesMapped” if reads were mapped to the human genome or to the species-specific genome, respectively. In the file name, “EachSpeciesUnique” indicates differential gene expression information for the genes initially flagged by the UpSet plot as being “uniquely” differentially expressed in response to poly(I:C) in a single NHP species compared to the response in human. Similarly, the other files give the same information for genes flagged as “unique” to “AllMonkeys” (squirrel monkey, rhesus macaque, pigtailed macaque, olive baboon); “OWMsOnly” (Old World Monkeys only; rhesus macaque, pigtailed macaque, olive baboon); or “CommonAllSpecies” (genes found in all the NHP species whose expression in response to poly(I:C) differed to that of human). 
