# Supplementary material for: Conservation of cell-intrinsic immune responses in diverse nonhuman primate species
Source: Life Sci Alliance. 2019 Oct 24;2(5):e201900495. doi: 10.26508/lsa.201900495 (PMC6814850; doi:10.26508/lsa.201900495)
Supplement: Supplementary file 11 [file LSA-2019-00495_Supplemental_Data_7.zip › DatasetS7/README_DatasetS7.rtf]

Here we compared our differential gene expression (DGE) profiles with those of Hagai et al. Nature 2018.We examined the DGEs we had in common with Hagai et al. - mouse, rhesus, and human — as indicated in the file name. In the case of rhesus, we show the DGE profiles for both genome mapping methods (indicated in file names by “HumanMapped” or “SpeciesMapped”, respectively). For either mapping method, the genes had been filtered to only those that a one-to-one human ortholog in rhesus. The human DGEs were limited to this same set of genes, hence the word “related” in the file name. We subsequently limited the Hagai et al. DGE profiles to these same genes for the rhesus and human analyses to make for a fairer comparison. For the mouse samples, we mapped the reads to the mouse genome and did not do any ortholog limitation of these genes. We did “translate” using ENSEMBL annotations the human ENSEMBL IDs given by Hagai et al. for each of their genes to the “equivalent” murine ENSEMBL ID. With the resultant gene sets, we then determined the overlap between our and Hagai et al.’s dataset.  Genes qualified as overlapping if they were log2FoldChange >= 2 in both sets and had a padj <= 0.05. 
