# Supplementary material for: Conservation of cell-intrinsic immune responses in diverse nonhuman primate species
Source: Life Sci Alliance. 2019 Oct 24;2(5):e201900495. doi: 10.26508/lsa.201900495 (PMC6814850; doi:10.26508/lsa.201900495)
Supplement: Supplementary file 12 [file LSA-2019-00495_Supplemental_Data_8.zip › DatasetS8/README_DatasetS8.rtf]

Here we compared our differential gene expression (DGE) profiles with the so-called “Core ISGs” identified in Shaw et al. 2017 in PLoS Biology. We examined the DGEs resulting from either the human or the species-specific genome mapping methods of the NHP species (indicated in file names by “HumanMapped” or “SpeciesMapped”, respectively). For either mapping method, the genes had been filtered to only those that a one-to-one human ortholog on a species-by-species basis for each of the NHP species. Thus, not all of the “Core ISGs” from Shaw et al. could be used in our comparison because they did not have a one-to-one human ortholog for the species we examined, but it was still >90% of the 62 genes. We used the same cut-offs as Shaw et al. in determining whether something was considered up-regulated (padj <= 0.05, log2FoldChange >= 2), hence the word “significant” used in the file name.For the mouse samples, the analysis was complicated by the fact that Shaw et al. gave their “Core ISGs” as the gene symbol which is not necessarily a unique identifier and for which in mouse there are sometimes multiple orthologs for the given human gene symbol. We thus first took their list of gene symbols and used our table of human orthologs for mouse genes generated from ENSEMBL and pulled the murine ENSEMBL IDs out. We then limited our DGE profile for mouse with this list of murine ENSEMBL IDs and once again set the level of significance to match Shaw et al.’s. 
