# Supplementary material for: Conservation of cell-intrinsic immune responses in diverse nonhuman primate species
Source: Life Sci Alliance. 2019 Oct 24;2(5):e201900495. doi: 10.26508/lsa.201900495 (PMC6814850; doi:10.26508/lsa.201900495)
Supplement: Supplementary file 13 [file LSA-2019-00495_Supplemental_Data_9.zip › DatasetS9/README_DatasetS9.rtf]

These are the log2FoldChange and padj values for the genes in NHP species that were found to be in the top 500 most significantly differentially expressed genes after using the human genome mapping approach (file name includes “HumanTop500Sig”) versus those after using the species-specific genome mapping approach (file name includes “SpeciesTop500Sig”). In each file, the padj  and log2FoldChange values are shown for the same genes but by the other mapping approach. These are the data that were used to generate Figure S11. 
